# Design, Synthesis and Biological Evaluation of New Pyrimidine Derivatives as Anticancer Agents

**DOI:** 10.3390/molecules26030771

**Published:** 2021-02-02

**Authors:** Valentina Noemi Madia, Alice Nicolai, Antonella Messore, Alessandro De Leo, Davide Ialongo, Valeria Tudino, Francesco Saccoliti, Daniela De Vita, Luigi Scipione, Marco Artico, Samanta Taurone, Ludovica Taglieri, Roberto Di Santo, Susanna Scarpa, Roberta Costi

**Affiliations:** 1 Istituto Pasteur-Fondazione Cenci Bolognetti, Dipartimento di Chimica e Tecnologie del Farmaco, “Sapienza” Università di Roma, p.le Aldo Moro 5, 00185 Rome, Italy; valentinanoemi.madia@gmail.com (V.N.M.); alessandro.deleo@uniroma1.it (A.D.L.); ialongo.1679357@studenti.uniroma1.it (D.I.); valeria.tudino@uniroma1.it (V.T.); luigi.scipione@uniroma1.it (L.S.); roberto.disanto@uniroma1.it (R.D.S.); roberta.costi@uniroma1.it (R.C.); 2 Department of Sensory Organs, “Sapienza” University of Rome, Viale del Policlinico 155, 00161 Rome, Italy; alice.nicolai@uniroma1.it (A.N.); marco.artico@uniroma1.it (M.A.); samanta.taurone@uniroma1.it (S.T.); 3 Department of Experimental Medicine, “Sapienza” University of Rome, Viale Regina Elena 324, I-00161 Rome, Italy; ludovica.taglieri@yahoo.it (L.T.); susanna.scarpa@uniroma1.it (S.S.); 4 D3 PharmaChemistry, Italian Institute of Technology, Via Morego 30, I-16163 Genova, Italy; francesco.saccoliti@iit.it; 5 Department of Environmental Biology, “Sapienza” University of Rome, p.le Aldo Moro 5, I-00185, Rome, Italy; daniela.devita@uniroma1.it

**Keywords:** pyrimidine, microwave reactions, breast cancer, glioblastoma multiforme, lung cancer, colon carcinoma

## Abstract

Background: Anticancer drug resistance is a challenging phenomenon of growing concern which arises from alteration in drug targets. Despite the fast speed of new chemotherapeutic agent design, the increasing prevalence of this phenomenon requires further research and treatment development. Recently, we reported a new aminopyrimidine compound—namely RDS 344—as a potential innovative anticancer agent. Methods: Herein, we report the design, synthesis, and anti-proliferative activity of new aminopyrimidine derivatives structurally related to RDS 3442 obtained by carrying out substitutions at position 6 of the pyrimidine core and/or on the 2-aniline ring of our hit. The ability to inhibit cell proliferation was evaluated on different types of tumors, glioblastoma, triple-negative breast cancer, oral squamous cell carcinomas and colon cancer plus on human dermal fibroblasts chosen as control of normal cells. Results: The most interesting compound was the *N*-benzyl counterpart of RDS 3442, namely **2a**, that induced a significant decrease in cell viability in all the tested tumor cell lines, with EC_50_s ranging from 4 and 8 μM, 4–13 times more active of hit. Conclusions: These data suggest a potential role for this class of molecules as promising tool for new approaches in treating cancers of different histotype.

## 1. Introduction

During the last five decades, since the launch of the “war on cancer” in 1971 in the United States, many scientists and pharmaceutical companies have been striving to conquer cancer [1]. Although the continuous development of new molecules in the treatment of cancer disease continues to increase, especially in terms of anticancer drugs, many of them do not satisfy the expectations of conquering cancer [2]. Indeed, the complex and heterogeneous nature of cancer is still associated with high mortality and morbidity rates [3] and drug resistance continues to be the principal limiting factor to achieve cures [4]. Resistance to treatment with anticancer drugs depends on several factors including individual variations in patients and somatic cell genetic differences in tumors, even those from the same tissue of origin. Principal mechanisms of drug resistance in cancer chemotherapy may include one or more altered energy-dependent membrane transporters that detect and eject anticancer drugs from cells (for example, the overexpression of the P-glycoprotein), altered target enzyme, decreased drug activation, increased drug degradation due to altered expression of drug-metabolizing enzymes, drug inactivation due to conjugation with increased glutathione, subcellular redistribution, drug interaction, enhanced DNA repair and failure to apoptosis as a result of mutated cell cycle proteins such as p53. [2,5]. For the above reasons, standard approaches are not enough to treat some tumor types, such as glioblastoma [6], triple-negative breast cancer [7], oral squamous cell carcinoma [3], lung [8] and colon cancer [9]. Among them, a major challenge is the treatment of head and neck squamous cell carcinomas (HNSCCs), including the tongue squamous cell carcinoma and pharynx squamous cell carcinoma. The group of HNSCC is the sixth most frequent cancer worldwide, with a global incidence of more than half a million annual cases [3] and about 65,000 of new cases only in the United States in 2019 [10], and it is highly resistant to a wide range of structurally different drugs with diverse cytotoxic mechanisms of action [3]. Indeed, the efficacy of pharmacological treatment is limited as patients acquire drug resistance, showing poor response to chemotherapeutics and therefore pushing physicians to use radiations and surgical interventions that often lead to a permanent impairment of oral functions [11,12]. Thus, despite many drugs have demonstrated promising ability, the development of new therapeutic options is still urgently needed.

**Figure 1:**
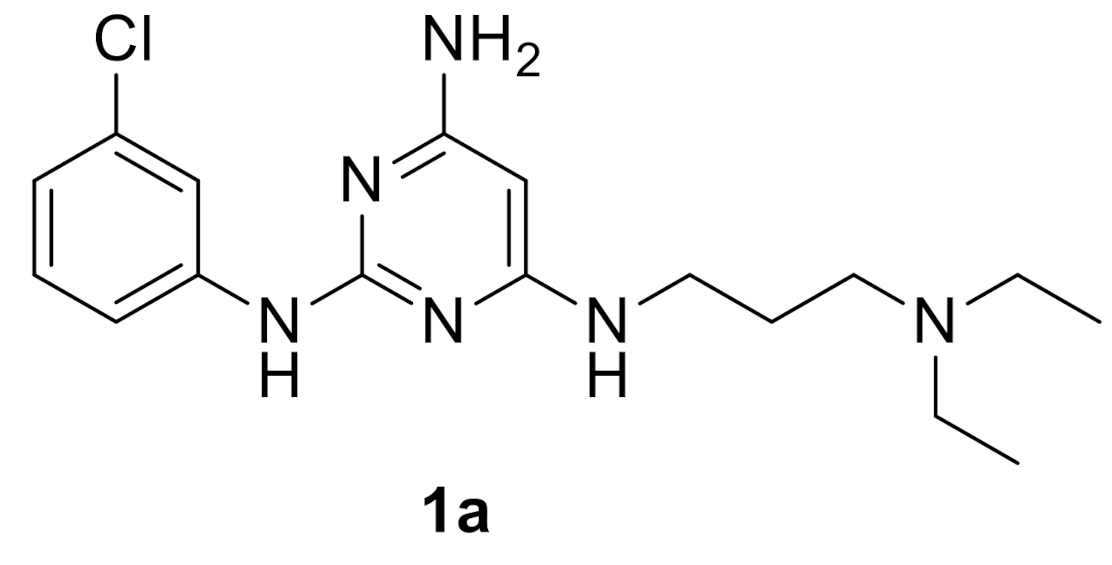
Structure of our previously reported anticancer aminopyrimidine derivative RDS 3442 (**1a**).

Recently, we synthesized and reported a new aminopyrimidine compound as a potential innovative anticancer agent namely RDS 3442 (**1a**, Figure 1) [13]. Notably, this compound was evaluated on three different human cancer types chosen on the basis of their unsatisfactory therapeutic strategies and poor prognosis: glioblastoma multiforme, triple-negative breast cancer and colon adenocarcinoma [14,15]. We demonstrated that compound **1a** is a potent inhibitor of replication, a negative regulator of cell cycle progression and an inducer of apoptosis for human cancer cells of different histotypes. Indeed, compound **1a** led to the upregulation of p21 and p27 and blockage of the cell cycle at G0/G1 at lower concentration (20 μM), while it induced apoptosis at higher concentrations (30–50 μM).

**Figure 2:**
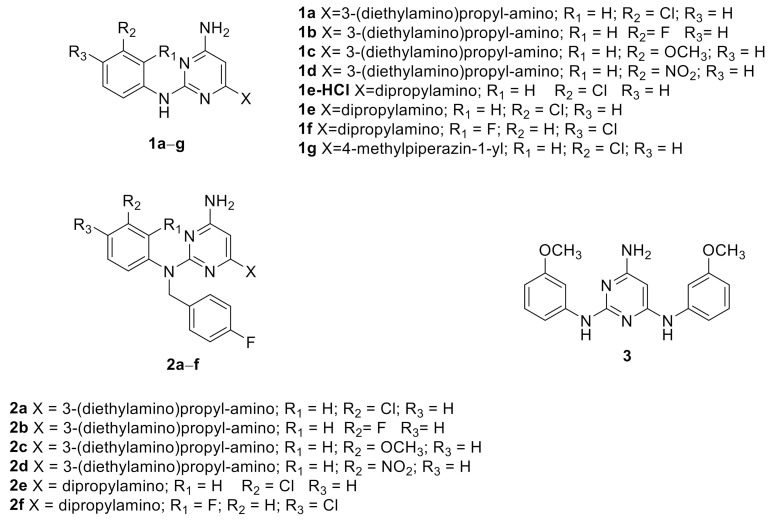
Structures of the newly designed aminopyrimidine derivatives **1a**–**g**, **2a**–**f** and **3**.

Taking into account these encouraging results, we designed and synthesized new aminopyrimidine derivatives structurally related to compound **1a** and evaluated their anti-proliferative activity on different cancer cell lines. All the newly designed compounds **1b**–**g**, **2a**–**f** and **3**, were conceived by carrying out substitutions in position 6 of the pyrimidine core and/or on the 2-aniline ring of RDS 3442 (Figure 2). Thus, by keeping fixed the structure of the reference compound, we investigated the role of the chlorine atom in position 3 of the aniline ring by substitution with both electron-withdrawing and electron-donating groups (fluorine, methoxy or nitro group), proper of compounds **1b**–**d**. Moreover, by derivatizing the 2-NH group of pyrimidine core with a *p*-fluorobenzyl ring, we obtained the corresponding alkylated derivatives **2a**–**d**. Differently, we substituted the *N*,*N*-diethylpropane-1,3-diamine in position 6 of reference compound with different alkylamines, in order to evaluate the effect of the nature of this substituent. Indeed, we synthesized compounds characterized by both secondary and tertiary amines as well as by linear and cyclic ones, obtaining derivatives **1e**–**g**. Moreover, by applying a similar approach to the one previously described, we synthesized also the corresponding alkylated derivatives, namely **2e** and **2f**. Notably, compound **3** was obtained as by-product from the nucleophilic aromatic substitution between the *m*-anisidine and the dichloropyrimidine core. Thanks to the quantifiable amount obtained, we decided to test also this compound in order to evaluate the effect of an additional aromatic amine on antitumoral activity.

## 2. Results and Discussion

### 2.1. Chemistry

**Scheme 1:**
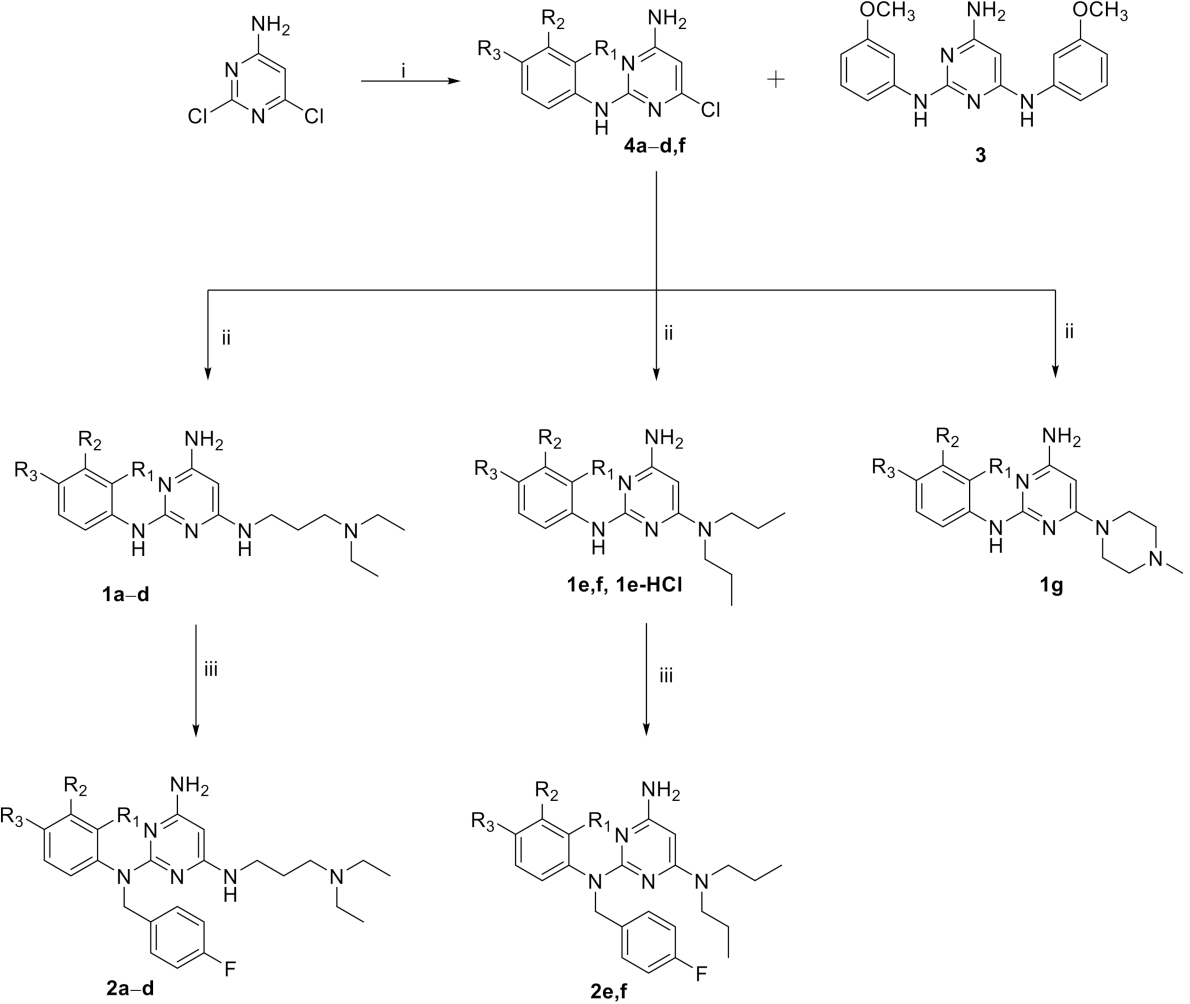
Synthetic route to **1a**–**g**, **2a**–**f** and **3** derivatives. *Reagents and Conditions*: Appropriate aniline, 2-methoxyethanol, reflux, overnight, 37–75% yield; ii: *N*^1^*,N*^1^-diethylpropane-1,3-diamine, K_2_CO_3_ *dry*, DMF *dry*, MW, 150 °C, 30 min, 33–42% yield for **1a** and **1b** or appropriate amine, DIPEA, *i*-PrOH, MW, 150 °C, 3 h, 23–57% yield for **1c**,**d**,**f**,**g** or dipropylamine, EtOH, MW, 190 °C, 3 h, 90–100% yield for compounds **1e-HCl** and **1e** (see Materials and Methods section) iii: 4-fluorobenzyl bromide, NaH 60% or Cs_2_CO_3_ for compound **2c**, DMF *dry,* 0 °C to rt, 0.5–5 h, 20–65% (see Materials and Methods section).

The synthesis of the tested compounds **1a**–**g**, **2a**–**f** and **3** is outlined in Scheme 1. Firstly, the intermediates **4a**–**d**,**f** were synthesized by reacting 4-amino-2,6-dichloropyrimidine with the appropriate aniline in 2-methoxyethanol and performing the reaction at reflux for 15 h [13]. Substitution with *m*-anisidine as reagent under the same conditions, gave both the 2- and 2,6-disubstituted pyrimidines **3** and **4c**, which were separable by chromatography. The corresponding 6-amino derivatives **1a** and **1b**–**g** were synthesized by microwave reactions in the presence of the appropriate amine reagents. In particular, derivatives **1a** and **1b** were synthesized in the presence of *N*^1^*,N*^1^-diethyl-propane-1,3-diamine by using anhydrous K_2_CO_3_ in anhydrous *N*,*N*-dimethylformamide (DMF) at 150 °C for 30 min [13], while compounds **1c**,**d** and **1f**,**g** were synthesized in the presence of the appropriate amine by using *N*,*N*-diisopropylethylamine (DIPEA) and isopropanol as solvent, using microwave irradiation [15]. The propanediamine-substituted compound **1e-HCl** was synthesized using microwave irradiation and isolated as the hydrochloride salt. The free base **1e** was obtained by dissolving the hydrochloride **1e-HCl** in water, adding an excess of sodium bicarbonate and extracting with chloroform. Lastly, the *N*-benzyl derivatives **2a**–**f** were obtained by alkylation in anhydrous DMF, with *p*-fluorobenzyl bromide in the presence of NaH (60% dispersion in mineral oil) as base for compounds **2a**,**b**,**d**–**f** or cesium carbonate for compound **2c**. The detailed synthetic procedures, the analytical and spectroscopic data of the synthesized compounds are reported in the Materials and Methods section.

The detailed structures of newly synthesized compounds **1b**–**g**, **2a**–**f** and **3** are listed in Table 1.

### 2.2. In Vitro Proliferation Assay

The possible antitumoral activity of these new compounds was analyzed by the effects exerted on the proliferation of five human tumor cell lines of different histotype. Therefore, the modifications on cell viability and replication determined by the treatment with each single compound were evaluated. The following human tumor cell lines triple-negative breast cancer MDA-MB231, colon carcinoma HT-29, glioblastoma multiforme U-87 MG, tongue squamous cell carcinoma CAL27 and pharynx squamous cell carcinoma FaDu were treated for 24 and 48 h with 10, 20, 30, 40, 50 and 60 μM of each single compound. Whenever the compound showed an important effect in inhibiting cell proliferation, other cell replication tests were performed at lower different concentrations (100 nM, 1 μM and 5 μM) [13].

When a compound demonstrated significant effects on cell replication inhibition, the proliferation assay was established also on human dermal fibroblasts (HF) at the same concentrations shown to be effective on tumor cells and the CC_50_ at 48 h was calculated and reported, in order to evaluate a possible cytotoxicity in somatic not transformed cells. When a compound did not exert any significant anti-proliferative activity at acceptable concentrations (higher than 30 μM), the proliferation assay was not performed on HF, because useless.

The median values from three different experiments of proliferation assays of all compounds for each cell line were evaluated and the EC_50_ at 24 and 48 h were calculated and reported. Anti-proliferative activities are reported in Table 2 (compounds **1b**–**g**, **3**) and Table 3 (compounds **2a**–**f**) in comparison with reference compound **1a**.

In general, derivatives of series 2 caused significant negative regulation of cell proliferation for all five tumors, showing higher potencies than series 1, even with a slight cytotoxic activity for normal fibroblasts, anyway completely not significant when considered the concentrations required for antitumor activity.

Indeed, among the newly synthesized compounds the most potent derivative proved to be **2a** that reported EC_50_s = 10–26 μM at 24 h of treatment and 5–8 μM at 48 h of treatment. In particular, within series 2, four of six compounds proved to be active at concentrations lower than 20 μM against all the tested cell lines at 48 h of treatment, differently from compounds of series 1 of which only **1b** was active at 20 μM on CAL27 at 48 h of treatment. Furthermore, the benzylated compounds proved to be generally endowed with higher potencies in respect to the hit compound. Moreover, it is possible to notice that compounds of both series 1 and 2 cause a more pronounced decrease in cell viability in HT-29 and CAL27 as compared with the other cell lines. Regarding derivative **3**, it proved to be active at concentrations up to 20 μM at 48 h of treatment, in the same range of compounds of series 2.

As regards series 1, the best acting compound was **1e** that reported EC_50_ values ranging from 25 and 45 μM at 48 h of treatment (only in the case of CAL27 a slightly higher potency in decreasing viability was exerted by derivative **1b**), without exerting cytotoxic activity on normal somatic cells up to 60 μM. Notably, compound **1e** showed higher potencies in respect to the hit compound **1a** against the tested cell lines. The hydrochloride salt **1e-HCl** showed in general comparable activity to that of its corresponding free base **1e**, suggesting that the salification did not lead to an improvement of potency in decreasing cell viability. 

Among the compounds of series 1, propanediamine substituted derivatives were characterized by different substituents in *meta* position of the aniline portion whose activity decreases in the following order: F > NO_2_ > Cl ≈ OCH_3_ (proper of derivatives **1b**, **1d**, **1a** and **1c**, respectively). In particular, compound **1c** proved to be completely inactive against all the tested cell lines up to 60 μM, with the sole exception of HT-29 and CAL-29 (EC_50_ = 57 μM). These results indicate the importance of an electron withdrawing substituent in *meta* position of the aniline residue. Differently, as regards dipropylamine substituted derivatives of series 1, it is possible to notice that the introduction of a *m*-Cl substituent on the aniline portion led to the best acting compound **1e**, while the *o*-Cl,*p*-F disubstitution led to less promising compound **1f** against all the tested cell lines (**1e**, EC_50_s = 25–45 μM; **1f**, EC_50_s = 50–75 μM). Lastly, the methylpiperazine derivative **1g** led to a less effective decrease in the cell viability of tumor cells in respect to both its dipropylamine substituted counterpart **1e** and its propanediamine substituted analog **1a**. These results indicate that, for compounds bearing a *m*-Cl substituent on the aniline residue, the activity decreases in the following order with respect to the 6-amino group: dipropylamine > propanediamine > methylpiperazine (corresponding to derivatives **1e**, **1a**, and **1g**, respectively).

**Figure 3:**
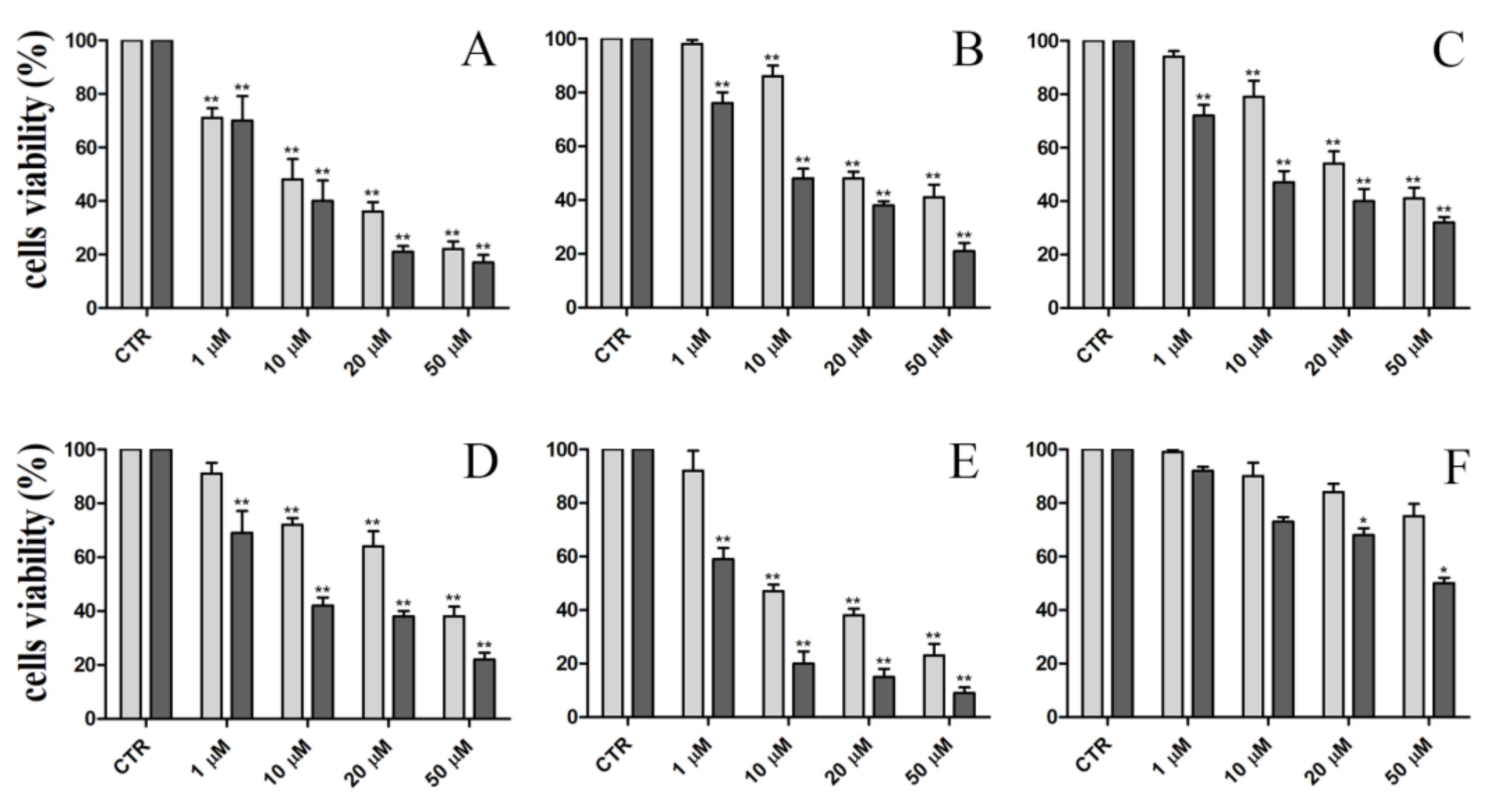
Cell viability of HT-29 (**A**), MDA-MB231 (**B**), U-87 MG (**C**), FaDu (**D**), CAL 27 (**E**), HF (**F**) untreated (CTR) and treated with compound **2a** at 1, 10, 20, 50 μM for 24 h (light gray) and 48 h (dark gray). The values are expressed as percentages of alive cells ± SD. * *p* < 0.01; ** *p* < 0.001.

As regards *N*-benzyl derivatives of series 2, the best acting compound was **2a** that reported EC_50_ values ranging from 10 to 26 μM at 24 h of treatment and from 5 to 8 μM at 48 h of treatment. Notably, compound **2a** proved to be from 4- to 13-fold more active than its *N*-benzyl analog **1a** against all the tested cell lines at 48 h of treatment. The data regarding the effects induced by compound **2a** on the proliferation decrease are represented in a bar chart (Figure 3). The activity of compound **2a** on the negative regulation of growth is significant at various concentrations and times for each tumor cell line (Figure 3A–E), while it is only weakly significant and exclusively at high concentrations at 48 h for normal fibroblasts (Figure 3F).

In general, compounds of series 2 caused a more significant decrease in viability of HT-29 and CAL27 in respect to the other cell lines. Among this series, propanediamine substituted derivatives were characterized by different substituents in *meta* position of the aniline portion whose activity decreases in the following order: Cl > F > NO_2_ ≈ OCH_3_ (proper of derivatives **2a**, **2b**, **2d** and **2c**, respectively). These data indicate the importance of an electron withdrawing group in the *meta* position of the aniline residue, similarly to that observed for propanediamine derivatives of series 1. As regards the amine in 6-position of the pyrimidine core, by substituting the propanediamine group with a dipropylamine one, a loss of activity was observed. Indeed, the dipropylamine substituted compounds **2e** and **2f** proved to be totally inactive or weakly active only against two cell lines, respectively (**2e**, EC_50_s > 60 μM vs. all cell lines; **2f**, EC_50_ on HT-29 = 30 μM, EC_50_ on CAL27 = 45 μM, EC_50_s > 60 μM vs. the other cell lines).

Regarding compound **3**, the introduction of two aromatic amines on the pyrimidine core showed promising results. Indeed, derivative **3** reported a more effective decrease in cell viability against all the tested cell lines in respect both to compounds of series 1 and to hit RDS 3442, showing also to be active in the same range of *N*-benzyl derivatives **2a**–**d** at both 24 h and 48 h of treatment. Moreover, among the *m*-OCH_3_ substituted derivatives, compound **3** proved to be endowed with higher potencies not only in respect to its propanediamine analog (**1c**) against all cell lines, but also in comparison with the ***N***-benzyl counterpart of **1c** (**2c**, EC_50_s = 15–35 μM) with the sole exception of FaDu, being **2c** about 2.5 folds more active than **3** at 48 h of treatment (**3**, EC_50_ = 42 μM; **2c**, EC_50_ = 18 μM). Interestingly, compound **3** did not prove to be cytotoxic on normal somatic cells up to 60 μM.

Overall, it can be stated that the introduction of the *p*-F benzyl ring on the nitrogen of the aniline residue led to a more significant decrease in cell viability for all the tested cell lines (*N*-benzyl substituted **2a** proved to be from 4- to 13-fold more active than its analog **1a**). Moreover, the propanediamine substituent in position 6 of the aminopyrimidine core can be replaced by a dipropylamine group, leading to an activity improvement. Anyway, this modification together with the introduction of a *N*-benzyl ring on the aniline residue is detrimental for the activity (compound **2e**, EC_50_s > 60 μM against all cell lines). Furthermore, the introduction of two aromatic amines on the aminopyrimidine core, proper of compound **3**, led to promising antitumor effects (**3** showed a more significant decrease in cell viability in respect both to derivatives of series 1 and to the hit compound, being also active in the same range of *N*-benzyl derivatives **2a**–**d**).

## 3. Materials and Methods 

### 3.1. Chemistry

#### 3.1.1. General Instrumentation

Melting points were determined on a Stuart SMP1 melting point apparatus (Bibby Scientific, Stone, UK) and are uncorrected. Compound purity was always >95% as determined by combustion analysis. Analytical results agreed to within ±0.40% of the theoretical values. IR spectra were recorded on a Spectrum-One spectrophotometer (Perkin Elmer, Shelton, CT, USA). ^1^H-NMR spectra were recorded at 400 MHz on a AC 400 Ultrashield 10 spectrophotometer (Bruker, Billerica, MA, USA). Dimethyl sulfoxide-*d*_6_ 99.9% (CAS 2206-27-1), deuterochloroform 98.8% (CAS 865-49-6) and acetone-*d*_6_ 99.9% (CAS 666-52-4) of isotopic purity (Aldrich, St. Louis, MO, USA) were used. Column chromatography was performed on silica gel (Merck, Darmstadt, Germany; 70−230 mesh) and on aluminum oxide (Merck; 70–230 mesh). All compounds were routinely checked on TLC by using aluminum-baked silica gel plates (Fluka, Honeywell, Charlotte, NC, USA; DC-Alufolien Kieselgel 60 F_254_) or TLC aluminum oxide 60 F_254_ basic (Merck). Developed plates were visualized by UV light. Solvents were reagent grade and, when necessary, were purified and dried by standard methods. Concentration of solutions after reactions and extractions involved the use of rotary evaporator (Büchi, Flawil, Switzerland) operating at a reduced pressure (ca. 20 Torr). Organic solutions were dried over anhydrous sodium sulfate (Merck). All solvents were freshly distilled under nitrogen and stored over molecular sieves for at least 3 h prior to use. Analytical results agreed to within ±0.40% of the theoretical values.

#### 3.1.2. Microwave Irradiation Experiments

Microwave reactions were conducted using a CEM Discover system unit (CEM. Corp., Matthews, NC, USA). The machine consists of a continuous focused microwave-power delivery system with operator selectable power output from 0 to 300 W. The temperature of the contents of the vessel was monitored using a calibrated infrared temperature control mounted under the reaction vessel. All experiments were performed using a stirring option whereby the contents of the vessel are stirred by means of a rotating magnetic plate located below the floor of the microwave cavity and a Teflon-coated magnetic stir bar in the vessel.

#### 3.1.3. General Experimental Procedures

**General Procedure A (GP-A) for the Synthesis of Derivatives 4a–d,f.**

To a solution of commercially available 2,6-dichloropyrimidine-4-amine (30 mmol) in anhydrous 2-methoxyethanol (100 mL), a suitable aniline (30 mmol) was added, and the reaction was stirred vigorously under reflux overnight. The solvent was reduced under vacuum, and the crude product was diluted with chloroform (300 mL). The organic layer was washed with water (300 mL), 1N HCl (250 mL) and saturated NaCl solution (3 × 300 mL), dried over Na_2_SO_4_ and concentrated under reduced pressure. The raw product was purified by column chromatography on silica gel (dichloromethane: methanol 98:2 as eluent). For each derivative amount of starting material; yield (%); melting point; IR; ^1^H-NMR and elemental analysis are reported. The structures of compound **4a** and its regioisomer was confirmed by means of 2D-NMR (see Figures S1 and S2 in Supplementary Materials). 

**General Procedure B (GP-B) for the Synthesis of Derivatives 1a and 1b.**

A mixture of an appropriate 2-substituted pyrimidine **4a**–**b** (1 mmol), *N*^1^*,N*^1^-diethylpropane-1,3-diamine (1 mmol) and anhydrous K_2_CO_3_ (2.5 mmol) in anhydrous DMF (0.8 mL) was irradiated with microwave at 150 °C for 30 min. After cooling, chloroform was added (5 mL) and the precipitate that formed was filtered and washed with ethyl acetate (7 mL). The organic layers were combined and evaporated under reduced pressure. The raw was quenched with 1N HCl (8 mL) and washed with chloroform (10 mL), then 1N NaOH was added until pH = 14 was reached and the aqueous layer was extracted with chloroform (2 × 20 mL). The organic layer was dried over Na_2_SO_4_ and concentrated under reduced pressure to give a dark brown oil that was purified by column chromatography on aluminium oxide (chloroform/methanol 9:1 as eluent). For each derivative amount of starting material; yield (%); melting point; IR; ^1^H-NMR and elemental analysis are reported.

**General Procedure C (GP-C) for the Synthesis of Derivatives 1c,d,f,g.**

In a microwave vial, the appropriate 2-substitued pyrimidine **4a**,**c**,**d**,**f** (1 mmol) was dissolved in isopropanol (1 mL), DIPEA (1.2 mmol) and appropriate amine (1.1 mmol) was added. The vial was sealed and heated by microwave at 150 °C for 3 h. After cooling, chloroform was added (10 mL) and the organic layer was quenched with 1N HCl (8 mL). Then 1N NaOH was added until pH = 12 was reached and the aqueous layer was extracted with chloroform (2 × 20 mL). The organic layer was washed with brine (2 × 20 mL), dried over Na_2_SO_4_ and concentrated under reduced pressure to give a dark brown oil that was purified by column chromatography on aluminium oxide For each derivatives amount of starting material; eluent system; yield (%); melting point; IR; ^1^H-NMR and elemental analysis are reported.

**General Procedure D (GP-D) for the Synthesis of Derivatives 2a,b,d–f.**

To a well-stirred suspension of NaH 60% (0.562 mmol or 0.843 mmol for hydrochloride salt **1e-HCl**) in anhydrous DMF (2 mL) derivatives **1a**,**b**,**d**–**f** (0.281 mmol), and the 4-fluorobenzyl bromide (0.320 mmol) was added at 0 °C under argon atmosphere. The reaction was stirred for the proper time at room temperature and then quenched with crushed ice. The mixture was extracted with chloroform (3 × 4 mL) and the organic extracts were collected, washed with brine (4 × 2 mL), dried over Na_2_SO_4_ and evaporated at reduced pressure [16]. The crude products were purified by column chromatography (Al_2_O_3_ or SiO_2,_ see experimental section) yielding the pure derivative **2a**,**b**,**d**–**f**. For each derivative amount of starting material; time reaction; chromatographic system; yield (%); melting point; IR; ^1^H-NMR and elemental analysis are reported.

#### 3.1.4. Specific Procedures and Characterization

***N^2^*-(3-Chlorophenyl)-*N^4^*-(3-(diethylamino)propyl)pyrimidine-2,4,6-triamine (1a).** Synthesized as reported in literature [10]. Yield: 55% as a red oil. IR ν 3303 (NH), 2968 (NH), 2935 (NH_2_) cm^−1^; ^1^H-NMR (DMSO-*d*_6_) δ 0.93 (t, 6H, *J* = 8 Hz, CH_3_), 1.58–1.65 (m, 2H, CH_2_-*CH_2_*-CH_2_-N), 2.40–2.45 (m, 6H, N-*CH_2_*-CH_3_ and CH_2_-CH_2_-*CH_2_*-N), 3.20–3.25 (m, 2H, *CH_2_*-CH_2_-CH_2_-N), 5.14 (s, 1H, CH pyrimidine), 5.86 (s, 2H, NH_2_), 6.27 (bs, 1H, NH), 6.83 (d, 1H, *J_o_* = 8 Hz, Ar), 7.19 (t, 1H, *J*_0_ = 8 Hz, Ar), 7.39 (d, 1H, *J*_0_ = 8 Hz, Ar), 8.06 (s, 1H, Ar), 8.76 (s, 1H, NH). Anal. Calcd for C_17_H_25_ClN_6_: C, 58.53; H, 7.22; Cl, 10.16; N, 24.09%. Found: C, 58.55; H, 7.20; N, 24.11%.

***N^4^*-(3-(Diethylamino)propyl)-*N^2^*-(3-fluorophenyl)pyrimidine-2,4,6-triamine (1b).** Compound **1b** was prepared from **4b** (0.200 g, 0.83 mmol) by means of GP-B. Yield: 33% as an orange solid; 100–102 °C; IR ν 3300 (NH), 3201 (NH), 2969 (NH_2_) cm^−1^; ^1^H-NMR (DMSO-*d*_6_) δ 0.93 (t, 6H, *J* = 8 Hz, CH_3_), 1.60–1.67 (m, 2H, CH_2_-*CH_2_*-CH_2_-N), 2.42–2.47 (m, 6H, N-*CH_2_*-CH_3_ and CH_2_-CH_2_-*CH_2_*-N), 3.17–3.22 (m, 2H, *CH_2_*-CH_2_-CH_2_-N), 5.02 (s, 1H, CH pyrimidine), 5.81 (s, 2H, NH_2_), 6.55 (bt, 1H, NH), 6.57 (d, 1H, *J*_0_ = 8 Hz, Ar), 7.16 (q, 1H, *J*_0_ = 8 Hz, Ar), 7.39 (d, 1H, *J*_0_ = 8 Hz, Ar), 7.94 (s, 1H, Ar), 8.70 (s, 1H, NH). Anal. Calcd for C_17_H_25_FN_6_: C, 61.42; H, 7.58; F, 5.72; N, 25.28%. Found: C, 61.48; H, 7.48; F, 5.52; N, 25.30%.

***N^4^*-(3-(Diethylamino)propyl)-*N^2^*-(3-methoxyphenyl)pyrimidine-2,4,6-triamine (1c).** Compound **1c** was prepared from **4c** (0.200 g, 0.80 mmol) and *N^1^,N^1^*-diethylpropane-1,3-diamine by means of GP-C. Dichloromethane/methanol 9.5:0.5. Yield: 23% as an orange oil. IR ν 3303 (NH), 2968 (NH), 2935 (NH_2_) cm^−1^; ^1^H-NMR (DMSO-*d*_6_) δ 0.95 (t, 6H, *J* = 8 Hz, CH_3_), 1.59–1.66 (m, 2H, CH_2_-*CH_2_*-CH_2_-N), 2.42–2.45 (m, 6H, N-*CH_2_*-CH_3_ and CH_2_-CH_2_-*CH_2_*-N), 3.20–3.21 (m, 2H, *CH_2_*-CH_2_-CH_2_-N), 3.71 (s, 3H, OCH_3_) 5.00 (s, 1H, CH pyrimidine), 5.72 (bs, 2H, NH_2_), 6.36–6.39 (m, 2H, aniline C2-H and C6-H), 7.04 (t, 1H, *J*_0_ = 8 Hz, aniline C5-H), 7.27 (d, 1H, *J*_0_ = 8 Hz, aniline C4-H), 7.62 (bs, 1H, NH), 8.37 (bs, 1H, NH). Anal. Calcd for C_18_H_28_N_6_O: C, 62.76; H, 8.19; N, 24.40%. Found: C, 62.80; H, 8.25; N, 24.22% 

***N^4^*-(3-(Diethylamino)propyl)-*N^2^*-(3-nitrophenyl)pyrimidine-2,4,6-triamine (1d).** Compound **1d** was prepared from **4d** (0.200 g, 0.75 mmol) and *N^1^,N^1^*-diethylpropane-1,3-diamine by means of GP-C. Ethyl acetate/methanol 9:1. Yield: 44% as a red oil. IR ν 3305 (NH), 2968 (NH), 2934 (NH_2_) cm^−1^; ^1^H-NMR (DMSO-*d*_6_) δ 0.92 (t, 6H, *J* = 8 Hz, CH_3_), 1.59–1.66 (m, 2H, CH_2_-*CH_2_*-CH_2_-N), 2.40–2.45 (m, 6H, N-*CH_2_*-CH_3_ and CH_2_-CH_2_-*CH_2_*-N), 3.26–3.27 (m, 2H, *CH_2_*-CH_2_-CH_2_-N), 5.06 (s, 1H, CH pyrimidine), 5.81 (bs, 2H, NH_2_), 6.54 (bs, 1H, NH), 7.42 (t, 1H, *J*_0_ = 8 Hz, aniline C5-H), 7.65 (d, 1H, *J*_0_ = 8 Hz, aniline C6-H), 8.06 (d, 1H, *J*_0_ = 8 Hz, aniline C4-H), 8.97 (bs, 1H, aniline C2-H), 9.06(s, 1H, NH). Anal. Calcd for C_17_H_25_N_7_O_2_: C, 56.81; H, 7.01; N, 27.28%. Found: C, 57.00; H, 7.35; N, 26.95%.

**Synthesis of *N^2^*-(3-chlorophenyl)-*N^4^,N^4^*-dipropylpyrimidine-2,4,6-triamine hydrochloride (1e-HCl) and *N^2^*-(3-chlorophenyl)-*N^4^,N^4^*-dipropylpyrimidine-2,4,6-triamine (1e).** In a microwave vial, intermediate 4a (0.2 g, 0.78 mmol) was dissolved in ethanol (2 mL) and dipropylamine (0.39 g, 3.9 mmol) was added. The vial was sealed and heated by microwave at 190 °C for 3 h. The solvent was removed under vacuum and the residue was treated with ethyl acetate (2 mL) and 1M HCl (1 mL). The formation of a precipitate was appreciated; the solid was filtered off, washed with petroleum ether yielding pure product **1e-HCl** as a hydrochloride salt. Yield: 90% as a white solid; 223–225 °C; IR ν 3135 (NH), 2962 (NH_2_) cm^−1^; ^1^H-NMR (DMSO-*d*_6_): δ 0.92 (t, 6H, *J* = 8 Hz, CH_3_), 1.55–1.61 (m, 4H, CH_2_*CH_2_*CH_3_), 5.29 (s, 1H, pyrimidine, C5-H), 7.15 (t, 1H, aniline C5-H), 7.35–7.39 (m, 4H, aniline C2-H, C4-H and NH_2_), 7.88 (s, 1H, aniline C6-H) 10.20 (bs, 1H, NH), 11.42 (bs, 1H, NH∙HCl). Anal. Calcd for C_16_H_23_Cl_2_N_5_: C, 53.94; H, 6.51; Cl, 19.90; N, 19.66%. Found: C, 56.00; H, 6.35; Cl, 20.01; N, 19.95%. Compound **1e-HCl** (0.100 g, 0.28 mmol) of was dissolved in water, has been adding an excess of sodium bicarbonate and extracting with chloroform (2 × 20 mL). The organic layer was dried over Na_2_SO_4_ and concentrated under reduced pressure to give pure 1e. Yield: 100% as a white solid; 96–98 °C; IR ν 3350 (NH), 2967 (NH_2_) cm^−1^; ^1^H-NMR (DMSO-*d*_6_): δ 0.96 (t, 6H, *J* = 8 Hz, CH_3_), 1.58–1.67 (m, 4H, CH_2_*CH_2_*CH_3_), 3.34–3.37 (m, 4H, *CH_2_*CH_2_CH_3_), 5.17 (s, 1H, pyrimidine, C5-H), 5.88 (bs, 2H, NH_2_), 6.88 (dd, 1H, *J*_0_ = 8 Hz, *Jm* = 4 Hz, aniline C6-H), 7.23 (t, 1H, *J*_0_ = 8 Hz, aniline C5-H), 7.62 (d, 1H, *J*_0_ = 8 Hz, aniline C4-H), 8.14 (s, 1H, aniline, C2-H), 8.78 (s, 1H, NH). Anal. Calcd for C_16_H_22_ClN_5_: C, 60.08; H, 6.93; Cl, 11.08; N, 21.90%. Found: C, 61.08; H, 7.02; Cl, 11.28; N, 22.03%.

***N^2^*-(4-Chloro-2-fluorophenyl)-*N^4^,N^4^*-dipropylpyrimidine-2,4,6-triamine (1f).** Compound **1f** was prepared from **4f** (0.200 g, 0.73 mmol) and dipropylamine by means of GP-C. Dichloromethane/methanol 9.8:0.2; Yield: 40% as a yellow oil; IR ν 2963 (NH), 2932 (NH_2_) cm^−1^; ^1^H-NMR (DMSO-*d*_6_) δ 0.91 (t, 6H, *J* = 8 Hz, CH_3_), 1.53–1.62 (m, 4H, CH_2_-*CH_2_*-CH_3_), 3.31 (t, 4H, *J*_0_ = 8 Hz, *CH_2_*-CH_2_-CH_3_), 5.17 (s, 1H, pyrimidine, C5-H), 5.94 (bs, 2H, NH_2_), 7.19 (d, 1H, *J*_0_ = 8 Hz, aniline C5-H), 7.21 (d, 1H, *J*_0_ = 8 Hz, aniline C3-H), 7.70 (bs, 1H, NH), 8.25 (t, 1H, *J*_0_ = 10 Hz aniline C6-H). Anal. Calcd for C_16_H_21_ClFN_5_: C, 56.89; H, 6.27; Cl, 10.49; F, 5.62; N, 20.73%. Found: C, 56.79; H, 6.30; Cl, 10.35; F, 5.58; N, 21.03%.

***N^2^*-(3-Chlorophenyl)-6-(4-methylpiperazin-1-yl)pyrimidine-2,4-diamine (1g).** Compound **1g** was prepared from **4a** (0.200 g, 0.78 mmol) and 1-methylpiperazine by means of GP-C. Dichloromethane/methanol 9:1; Yield: 57% as a red oil. IR ν 3285 (NH), 2849 (NH_2_) cm^−1^; ^1^H-NMR (DMSO-*d*_6_) δ 2.20 (s, 3H, CH_3_), 2.35 (t, 4H, *J* = 16 Hz, piperazine), 5.25 (s, 1H, pyrimidine C5-H), 6.02 (bs, 2H, NH_2_), 6.83 (d, 1H, *J*_0_ = 8 Hz, aniline C6-H), 7.18 (t, 1H, *J*_0_ = 8 Hz aniline C5-H), 7.59 (d, 1H, *J*_0_ = 8 Hz aniline C4-H), 7.98 (s, 1H, aniline C2-H), 9.57 (s, 1H, NH). Anal. Calcd for C_15_H_19_ClN_6_: C, 56.51; H, 6.01; Cl, 11.12; N, 26.36%. Found: C, 56.62; H, 6.11; Cl, 11.20; N, 26.56%.

***N^2^*-(3-chlorophenyl)-N^4^-(3-(diethylamino)propyl)-*N^2^*-(4-fluorobenzyl)pyrimidine-2,4,6-triamine (2a).** Compound **2a** was prepared from **1a** (0.200 g, 0.57 mmol) by means of GP-D. Time of reaction: 2 h; Al_2_O_3_ and chloroform/methanol 9:1 as eluent; Yield: 47% as a red oil; IR ν 3322 (NH), 2968 (NH_2_) cm^−1^; ^1^H-NMR (DMSO-*d*_6_): δ 0.96 (t, 6H, *J* = 8 Hz, CH_3_), 1.59–1.62 (m, 2H, CH_2_-*CH_2_*-CH_2_-N), 2.39–2.45 (m, 6H, N-*CH_2_*-CH_3_ and *CH_2_*-CH_2_-CH_2_-N), 3.12–3.22 (m, 2H, *CH_2_*-CH_2_-CH_2_-N), 4.87 (s, 1H, pyrimidine C5-H), 5.18 (s, 2H, benzyl CH_2_), 5.84 (bs, 2H, NH_2_), 6.24 (bs, 1H, NH) 7.12–7.45 (m, 8H, aniline and benzyl H). Anal. Calcd for C_24_H_30_ClFN_6_: C, 63.08; H, 6.62; Cl, 7.76; F, 4.16; N, 18.39%. Found: C, 62.88; H, 6.51; Cl, 7.96; F, 4.26; N, 18.22%.

***N^4^*-(3-(diethylamino)propyl)-*N^2^*-(4-fluorobenzyl)-*N^2^*-(3-fluorophenyl)pyrimidine-2,4,6-triamine (2b).** Compound **2b** was prepared from **1b** (0.200 g, 0.60 mmol) by means of GP-D. Time of reaction: 2 h; Al_2_O_3_ and chloroform/methanol 9:1 as eluent; Yield: 35% as an orange solid; 86–88 °C; IR ν 3190 (NH), 2977 (NH_2_) cm^−1^; ^1^H-NMR (DMSO-*d*_6_): δ 0.95 (t, 6H, *J* = 8 Hz, CH_3_), 1.54–1.58 (m, 2H, CH_2_-*CH_2_*-CH_2_-N), 2.35–2.46 (m, 6H, N-*CH_2_*-CH_3_ and CH_2_-CH_2_-*CH_2_*-N), 3.07–3.12 (m, 2H, *CH_2_*-CH_2_-CH_2_-N), 5.04 (s, 1H, pyrimidine C5-H), 5.28 (s, 2H, benzyl CH_2_), 5.84 (bs, 2H, NH_2_), 6.44 (bs, 1H, NH) 7.10–7.34 (m, 8H, aniline and benzyl H). Anal. Calcd for C_24_H_30_F_2_N_6_: C, 65.43; H, 6.86; F, 8.63; N, 19.08%. Found: C, 65.53; H, 6.78; F, 8.58; N, 19.11%.

**Synthesis of *N^4^*-(3-(diethylamino)propyl)-*N^2^*-(4-fluorobenzyl)-*N^2^*-(3-methoxyphenyl)- pyrimidine-2,4,6-triamine (2c).** A solution of **1c** (0.200 g, 0.58 mmol) in anhydrous DMF (2 mL) was added of Cs_2_CO_3_ (0.283 g, 0.87 mmol) and the result solution was stirred for 10 min. 4-fluorobenzyl bromide (0.132 g, 0.696 mmol) was added. The reaction was stirred for 5 h at room temperature and then quenched with crushed ice. The mixture was extracted with chloroform (3 × 4 mL) and the organic extracts were collected, washed with brine (4 × 2 mL), dried over Na_2_SO_4_ and evaporated at reduced pressure. The crude material was purified by column chromatography on alumina oxide gel, dichloromethane/methanol 9:1 as eluent to give the pure derivative **2c**. Yield: 20% as a light yellow oil IR ν 3319 (NH), 2967 (NH_2_) cm^−1^; ^1^H-NMR (DMSO-*d*_6_): δ 0.88 (t, 6H, *J* = 8 Hz, CH_3_), 1.46–1.53 (m, 2H, CH_2_-*CH_2_*-CH_2_-N), 2.28–2.44 (m, 6H, N-*CH_2_*-CH_3_ and CH_2_-CH_2_-*CH_2_*-N), 3.01–3.05 (m, 2H, *CH_2_*-CH_2_-CH_2_-N), 3.67 (s, 1H, OCH_3_), 4.94 (s, 1H, pyrimidine C5-H), 5.17 (s, 2H, benzyl CH_2_), 5.71 (bs, 2H, NH_2_), 6.31 (bs, 1H, NH), 6.60 (dd, 1H, *J*_0_ = 8 Hz, *Jm*= 4 Hz, aniline C4-H), 6.77–6.81 (m, 2H, benzyl H), 7.03–7.14 (m, 3H, aniline C5-H and benzyl H), 7.26–7.28 (m, 2H, aniline C2-H and C6-H). Anal. Calcd for C_25_H_32_FN_6_O: C, 66.35; H, 7.35; F, 4.20; N, 18.57%. Found: C, 66.25; H, 7.39; F, 4.26; N, 18.43%.

**(*N^4^*-(3-(Diethylamino)propyl)-*N^2^*-(4-fluorobenzyl)-*N^2^*-(3-nitrophenyl)pyrimidine-2,4,6-triamine (2d).** Compound **2d** was prepared from **1d** (0.200 g, 0.56 mmol) by means of GP-D. Time of reaction: 30 min; Al_2_O_3_ and chloroform/methanol 9.5:0.5 as eluent; Yield: 47% as an orange solid; 158–160 °C; IR ν 3317 (NH), 2967 (NH_2_) cm^−1^; ^1^H-NMR (DMSO-*d*_6_): δ 0.93 (t, 6H, *J* = 8 Hz, CH_3_), 1.54–1.58 (m, 2H, CH_2_-*CH_2_*-CH_2_-N), 2.32–2.44 (m, 6H, N-*CH_2_-*CH_3_ and CH_2_-CH_2_-*CH_2_-*N), 3.08–3.13 (m, 2H, *CH_2_*-CH_2_-CH_2_-N), 5.09 (s, 1H, pyrimidine C5-H), 5.38 (s, 2H, benzyl CH_2_), 5.90 (bs, 2H, NH_2_), 6.53 (bs, 1H, NH), 7.13–7.17 (m, 2H, benzyl H), 7.33–7.39 (m, 2H, benzyl H), 7.55 (t, 1H, aniline C5-H), 7.74 (dd, 1H, *J*_0_ = 8 Hz, *J_m_* = 4 Hz, aniline C6-H), 7.88 (dd, 1H, *J*_0_ = 8 Hz, *J_m_* = 4 Hz, aniline C4-H), 8.26 (s, 1H, aniline C2-H). Anal. Calcd for C_24_H_30_FN_7_O_2_: C, 61.65; H, 6.47; F, 4.06; N, 20.97%. Found: C, 61.70; H, 6.51; F, 3.97; N, 21.02%.

***N^2^*-(3-Chlorophenyl)-*N^2^*-(4-fluorobenzyl)-*N^4^,N^4^*-dipropylpyrimidine-2,4,6-triamine (2e).** Compound **2e** was prepared from **1e-HCl** (0.200 g, 0.56 mmol) by means of GP-D. Time of reaction: 4.5 h; SiO_2_ and dichloromethane/methanol 9.5:0.5 as eluent; Yield: 56% as a light yellow oil; IR ν 2965 (NH), 2875 (NH_2_) cm^−1^; ^1^H-NMR (DMSO-*d*_6_) δ 0.73 (bt, 6H, CH_3_), 1.45 (m, 4H, CH_2_-*CH_2_*_-_CH_3_), 3.35 (bt, 4H, *CH_2_*-CH_2_-CH_3_), 4.85 (s, 1H, pyrimidine C5-H), 5.09 (s, 2H, benzyl CH_2_), 5.78 (bs, 2H, NH_2_), 7.07–7.39 (m, 8H, aniline and benzyl H). Anal. Calcd for C_23_H_27_ClFN_5_: C, 64.55; H, 6.36; Cl, 8.28; F, 4.44; N, 16.37%. Found: C, 64.85; H, 6.25; Cl, 8.32; F, 4.64; N, 15.98%.

***N^2^*-(4-Chloro-2-fluorophenyl)-*N^2^*-(4-fluorobenzyl)-*N^4^,N^4^*-dipropylpyrimidine-2,4,6-triamine (2f).** Compound **2f** was prepared from **1f** (0.200 g, 0.59 mmol) by means of GP-D. Time of reaction: 1.5 h; SiO_2_ and *n*-hexane/ethyl acetate 8:2 as eluent; Yield: 65% as a yellow oil; IR ν 3307 (NH), 2966 (NH_2_) cm^−1^; ^1^H-NMR (DMSO-*d*_6_) δ 0.74 (bt, 6H, CH_3_), 1.43–1.47 (m, 4H, 4H, CH_2_-*CH_2_*_-_CH_3_), 3.30 (bt, *CH_2_*-CH_2_-CH_3_), 4.65 (s, 1H, pyrimidine, C5-H), 4.97 (s, 2H, benzyl CH_2_), 5.77 (bs, 2H, NH_2_), 7.07–7.53 (m, 7H, aniline and benzyl H). Anal. Calcd for C_23_H_26_ClF_2_N_5_: C, 61.95; H, 5.88; Cl, 7.95; F, 8.52; N, 15.70%. Found: C, 61.94; H, 5.87; Cl, 7.96; F, 8.51; N, 15.69%.

***N^2^,N^4^*-bis(3-Methoxyphenyl)pyrimidine-2,4,6-triamine (3).** Compound **3** was prepared from *m*-anisidine (3.0 g, 24.36 mmol) by means of GP-A. Yield: 16% as a brown oil; IR ν 3363 (NH), 2932 (NH_2_) cm^−1^; ^1^H-NMR (DMSO-*d*_6_) δ 3.68 (s, 3H, OCH_3_), 3.71 (s, 3H, OCH_3_), 5.39 (s, 1H, pyrimidine C5-H), 6.07 (bs, 2H, NH_2_), 6.41 (d, 1H, *J*_0_ = 8 Hz, aniline C6-H), 6.48 (d, 1H, *J*_0_ = 8 Hz, aniline C6′-H), 7.05–7.15 (m, 4H, aniline C2-H, C2′H, C4′H and C5′H), 7.18 (d, 1H, *J*_0_ = 8 Hz, aniline C4-H), 7.35 (s, 1H, aniline C2-H), 8.57 (s, 1H, NH), 8.65 (s, 1H, NH). Anal. Calcd for C_18_H_19_N_5_O_2_: C, 64.08; H, 5.68; N, 20.76; O, 9.48%. Found: C, 64.10; H, 5.65; N, 20.80%.

**6-Chloro-*N^2^*-(3-chlorophenyl)pyrimidine-2,4-diamine (4a).** The synthesis of compound **4a** is reported in the literature [10]. Yield: 43% as a white solid; 103–105 °C; IR ν 3385 (NH), 3103 (NH_2_) cm^−1^; ^1^H-NMR (DMSO-*d*_6_) δ 5.95 (s, 1H, pyrimidine C5-H), 6.93 (d, 1H, *J*_0_ = 8 Hz, aniline C6-H), 7.03 (s, 2H, NH_2_), 7.24 (t, 1H, *J*_0_ = 8 Hz, aniline C5-H), 7.68 (d, 1H, *J*_0_ = 8 Hz, aniline C4-H), 7.88 (s, 1H, aniline C2-H), 9.55 (s, 1H, NH). Anal. Calcd for C_10_H_8_Cl_2_N_4_: C, 47.08; H, 3.16; Cl, 27.80; N, 21.96%. Found: C, 47.18; H, 3.25; Cl, 27.90; N, 22.02%.

**6-Chloro-*N^2^*-(3-fluorophenyl)pyrimidine-2,4-diamine (4b).** Compound **4b** was prepared from 3-fluoroaniline (3.0 g, 27.0 mmol) by means of GP-A. Yield: 75% as a yellow solid; 133–135 °C; IR ν 3395 (NH), 3120 (NH_2_) cm^−1^; ^1^H-NMR (DMSO-*d*_6_) δ 5.97 (s, 1H, pyrimidine C5-H), 6.93 (d, 1H, *J*_0_ = 8 Hz, aniline C6-H), 7.05 (s, 2H, NH_2_), 7.24 (t, 1H, *J*_0_ = 8 Hz, aniline C5-H), 7.68 (d, 1H, *J*_0_ = 8 Hz, aniline C4-H), 7.88 (s, 1H, aniline C2-H), 9.56 (s, 1H, NH). Anal. Calcd for C_10_H_8_FClN_4_: C, 50.33; H, 3.38; Cl, 14.86; F, 7.96; N, 23.48%. Found: C, 50.45; H, 3.35; Cl, 15.00; F, 8.02; N, 23.02%.

**6-Chloro-*N^2^*-(3-methoxyphenyl)pyrimidine-2,4-diamine (4c).** Compound **4c** was prepared from *m*-anisidine (3.0 g, 24.36 mmol) by means of GP-A. Yield: 53% as an orange oil; IR ν 3365 (NH), 3093 (NH_2_) cm^−1^; ^1^H-NMR (DMSO-*d*_6_) δ 3.80 (s, 3H, -OCH_3_), 6.00 (s, 1H, pyrimidine C5-H), 6.57 (dd, 1H, *J*_0_ = 8 Hz, *J_m_* = 4 Hz, aniline C4-H), 7.02 (s, 2H, NH_2_), 7.20 (t, 1H, *J*_0_ = 8 Hz, aniline C5-H), 7.34 (d, 1H, *J*_0_ = 8 Hz, aniline C6-H), 7.56 (s, 1H, aniline C2-H), 9.39 (s, 1H, NH). Anal. Calcd for C_11_H_11_ClN_4_O: C, 52.70; H, 4.42; Cl, 14.14; N, 22.35%. Found: C, 52.77; H, 4.32; Cl, 14.24; N, 22.15%.

**6-Chloro-*N^2^*-(3-nitrophenyl)pyrimidine-2,4-diamine (4d).** Compound **4d** was prepared from 3-nitroaniline (3.0 g, 21.72 mmol) by means of GP-A. Yield: 45% as a yellow solid; 228–230 °C; IR ν 3388 (NH), 3113 (NH_2_) cm^−1^; ^1^H-NMR (DMSO-*d*_6_) δ 6.08 (s, 1H, pyrimidine C5-H), 7.14 (bs, 2H, NH_2_), 7.59 (t, 1H, *J*_0_ = 8 Hz, aniline C5-H), 7.82 (dt, 1H, *J*_0_ = 8 Hz, *J_m_* = 4 Hz, aniline C4-H), 8.25 (dt, 1H, *J*_0_ = 8 Hz, *J_m_* = 4 Hz, aniline C6-H), 8.72 (t, 1H, *J_m_* = 4 Hz, aniline C2-H), 9.85 (s, 1H, NH). Anal. Calcd for C_10_H_8_ClN_5_O_2_: C, 45.21; H, 3.04; Cl, 13.35; N, 26.36%. Found: C, 45.28; H, 3.09; Cl, 13.45; N, 26.26%.

**6-Chloro-*N^2^*-(4-chloro-2-fluorophenyl)pyrimidine-2,4-diamine (4f).** Compound **4f** was prepared from 4-chloro-2-fluoroaniline (3.0 g, 20.61 mmol) by means of GP-A. Yield: 37% as a grey solid; 111–112 °C; IR ν 3390 (NH), 3110 (NH_2_) cm^−1^; ^1^H-NMR (DMSO-*d*_6_) δ 5.97 (s, 1H, pyrimidine C5-H), 7.11 (d, 1H, *J*_0_ = 8 Hz, aniline C5-H), 7.03 (s, 2H, NH_2_), 7.36 (d, 1H, *J*_0_ = 8 Hz, aniline C6-H), 8.18 (t, 1H, *J*_0_ = 8 Hz, aniline C3-H), 9.65 (s, 1H, NH). Anal. Calcd for C_10_H_7_Cl_2_FN_4_: C, 43.98; H, 2.58; Cl, 25.96; F, 6.96; N, 20.52%. Found: C, 44.08; H, 2.78; Cl, 26.16; F, 6.76; N, 20.45%.

### 3.2. Biological Assays

#### 3.2.1. Cell Cultures and Treatments

The following human tumor cell lines were utilized in the present study: glioblastoma multiforme U-87 MG, triple-negative breast cancer MDA-MB231, colon carcinoma HT-29, tongue squamous cell carcinoma CAL27 and pharynx squamous cell carcinoma FaDu, all of which obtained from the American Type Culture Collection (Manassas, VA, USA). In addition, human dermal fibroblast primary cultures (HF) used as control of non-transformed cells, had been previously established in our laboratory [17].

The cells were grown at 37 °C and 5% CO_2_ in Dulbecco’s modified Eagle’s medium or RPMI 1640 medium supplemented with 10% fetal bovine serum, 2 mM glutamine and 50 U/mL penicillin-streptomycin (Sigma-Aldrich).

All compounds were solubilized in dimethylsulfoxide (DMSO) (Sigma) for a 10 mM stock solution and utilized to final concentrations from 100 nM to 60 μM for 24 and 48 h. Control cells were treated with equivalent amounts of DMSO in every experiment.

#### 3.2.2. Cytotoxicity Assay

To determine cytotoxicity, a sulforhodamine B colorimetric assay was performed. Cells (1.5 × 10^4^) were plated in a 96-well plate, grown for 24 h and then treated with different concentrations of each compound for 24 and 48 h at 37 °C. Cells were then fixed with 50% trichloroacetic acid for 1 h at 4 °C and stained for 30 min at room temperature with 0.4% sulforhodamine B in 1% acetic acid. Excess dye was removed by washing four times with 1% acetic acid. Protein-bound dye was dissolved in 10 mM Tris (pH 10), and optical density was determined at 510 nm using a microplate reader. Each experiment was performed three times and the media was calculated.

#### 3.2.3. Statistical Analysis

All results were analyzed using one-way analysis of variance, and significance was evaluated using Tukey’s honest significant difference post-hoc test.

## 5. Conclusions

The anticancer fight has utilized a broad and heterogeneous therapeutic armamentarium, allowing considerable progress so far, however, drug resistance among other factors still limits treatment efficacy. Therefore, the development of new effective anticancer agents is still a pressing open issue. In the light of the above, we followed up on our previous achievements obtained with the aminopyrimidine compound RDS 3442 that proved to be a potent inhibitor of replication, a negative regulator of cell cycle progression and an inducer of apoptosis for human cancer cells of different histotypes. Consequently, we synthesized a new series of aminopyrimidine derivatives structurally related to RDS 3442 by carrying out substitutions in position 6 of the pyrimidine core and/or on the 2-aniline ring. Structure-activity relationship studies allowed us to conclude that it is possible to achieve an activity improvement when the aminopyrimidine core is endowed with: (i) a *p*-F benzyl ring on the nitrogen of the aniline residue in 2-position along with a primary aliphatic base as the propanediamine one in position 6; or (ii) a secondary aliphatic base as the dipropylamine in 6-position without the *N*-benzyl ring linked to the aniline substituent in position 2. Indeed, the best compound proved to be the *N*-benzyl counterpart of RDS 3442, namely **2a**, that induced the highest rate of a significant decrease in cell viability in all the tested cell lines, with EC_50_ values ranging from 4 and 8 μM, 4–13 times more active of the hit. Although more investigations are needed to deepen the mechanism of action of this newly synthesized aminopyrimidines, the data obtained so far suggest a potential role for this class of molecules as promising tool for new approaches in treating various cancers.

## Figures and Tables

**Table 1:**
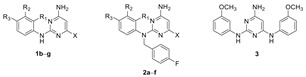
Detailed chemical structures of the newly synthesized compounds **1b**–**g**, **2a**–**f** and **3**.

Cpd	R_1_	R_2_	R_3_	X
1b	H	F	H	3-(diethylamino)propyl-amino
1c	H	OCH_3_	H	3-(diethylamino)propyl-amino
1d	H	NO_2_	H	3-(diethylamino)propyl-amino
1e-HCl	H	Cl	H	Dipropylamino
1e	H	Cl	H	Dipropylamino
1f	F	H	Cl	Dipropylamino
1g	H	Cl	H	4-methylpiperazin-1-yl
2a	H	Cl	H	3-(diethylamino)propyl-amino
2b	H	F	H	3-(diethylamino)propyl-amino
2c	H	OCH_3_	H	3-(diethylamino)propyl-amino
2d	H	NO_2_	H	3-(diethylamino)propyl-amino
2e	H	Cl	H	Dipropylamino
2f	F	H	Cl	Dipropylamino
3	-	-	-	-

**Table 2:** Cytotoxicity and antitumor activities of the newly synthesized compounds **1b**–**g** and **3** in comparison with reference compound **1a**.

Cpd	EC_50_ (μM) ^1^					CC_50_ (μM) ^2^
	HT-29	U-87 MG	MDA-MB231	CAL27	FaDu	HF
**1b**	29.8 (48 h) ± 3.2	47.3 (48 h) ± 3.6	35.7 (48 h) ± 3.2	20.3 (48 h) ± 1.8	43.4 (48 h) ± 3.9	88 (48 h) ± 9.0
**1c**	57 (48 h) ± 2.4	68 (48 h) ± 5.0	70.5 (48 h) ± 6.0	57 (48 h) ± 4.0	71 (48 h) ± 5.6	nd ^3^
**1d**	40.4 (48 h) ± 4.8	54.9 (48 h) ± 4.8	49.2 (48 h) ± 3.5	35.3 (48 h) ± 3.9	46.8 (48 h) ± 5.0	97 (48 h) ± 13.0
**1e-HCl**	29.3 (48 h) ± 1.7	36.6 (48 h) ± 2.9	50.2 (48 h) ± 4.5	30.3 (48 h) ± 2.4	39.8 (48 h) ± 4.0	76 (48 h) ± 5.5
**1e**	28.5 (48 h) ± 1.9	45.3 (48 h) ± 3.8	34.2 (48 h) ± 2.5	24.7 (48 h) ± 1.8	29.7 (48 h) ± 3.2	68 (48 h) ± 5.2
**1f**	50 (48 h) ± 3.3	75 (48 h) ± 4.9	58 (48 h) ± 6.1	55 (48 h) ± 5.7	67 (48 h) ± 4.9	98 (48 h) ± 8.0
**1g**	60 (48 h) ± 5.8	82 (48 h) ± 7.0	55 (48 h) ± 3.7	60 (48 h) ± 6.2	87 (48 h) ± 7.0	nd ^3^
**3**	28.2 (24 h) ± 1.4 11.7 (48 h) ± 0.9	22.9 (48 h) ± 2.5	10.2 (48 h) ± 1.5	20.3 (24 h) ± 2.8 10.4 (48 h) ± 0.8	42.1 (24 h) ± 3.6 19.7 (48 h) ± 2.0	65 (48 h) ± 4.1
**1a**	51.8 (48 h) ± 4.2	75.2 (48 h) ± 6.2	34.8 (48 h) ± 2.8	54.2 (48 h) ± 4.8	69.3 (48 h) ± 5.8	73.6 (48 h) ± 6.0

^1^ Half maximal effective concentration (μM) on five different cell lines at 24 and 48 h ± SD. ^2^ Half maximal cytotoxic concentration (μM). ^3^ not determined.

**Table 3:** Cytotoxicity and antitumor activities of the newly synthesized compounds **2a**–**f** in comparison with reference compound **1a**.

Cpd	EC_50_ (µM) ^1^					CC_50_ (µM) ^2^
	HT-29	U-87 MG	MDA-MB231	CAL27	FaDu	HF
**2a**	10.2 (24 h) ± 0.6 5.4 (48 h) ± 0.4	22.0 (24 h) ± 1.3 7.5 (48 h) ± 0.6	18.0 (24 h) ± 0.7 7.9 (48 h) ± 0.8	9.7 (24 h) ± 0.6 4.3 (48 h) ± 0.5	26.2 (24 h) ± 3.2 8.5 (48 h) ± 1.1	50.2 (48 h) ± 3.8
**2b**	20.2 (24 h) ± 1.9 10.4 (48 h) ± 0.8	17.7 (48 h) ± 1.2	7.3 (48 h) ± 0.5	5.1 (48 h) ± 0.3	23.8 (48 h) ± 2.1	54.7 (48 h) ± 4.2
**2c**	34.7 (24 h) ± 2.8 18.3 (48 h) ± 1.2	19.8 (48 h) ± 1.4	27.2 (48 h) ± 1.8	14.9 (48 h) ± 1.1	18.2 (48 h) ± 1.3	50.3 (48 h) ± 3.7
**2d**	25.2 (24 h) ± 2.0 12.7 (48 h) ± 1.1	30.1 (48 h) ± 2.4	17.3 (48 h) ± 1.9	10.3 (48 h) ± 0.9	20.5 (48 h) ± 2.3	55.2 (48 h) ± 4.9
**2e**	65.2 (48 h) ± 5.0	72.4 (48 h) ± 3.8	60.8 (48 h) ± 7.0	59.3 (48 h) ± 4.7	84.4 (48 h) ± 5.0	nd 3
**2f**	30.0 (48 h) ± 1.5	67.0 (48 h) ± 5.0	58.3 (48 h) ± 6.5	45.4 (48 h) ± 1.8	75.3 (48 h) ± 6.9	nd 3
**1a**	51.8 (48 h) ± 4.2	75.2 (48 h) ± 6.2	34.8 (48 h) ± 2.8	54.2 (48 h) ± 4.8	69.3 (48 h) ± 5.8	73.6 (48 h) ± 6.0

^1^ Half maximal effective concentration (μM) on five different cell lines at 24 and 48 h ± SD. ^2^ Half maximal cytotoxic concentration (μM). ^3^ not determined.

## Data Availability

Data is contained within the article.

## Supplementary Materials

The following are available online, Figure S1: NMR Spectra for **1b**; Figure S2: NMR Spectra for **1c**; Figure S3: NMR Spectra for **1d**; Figure S4: NMR Spectra for **1e**; Figure S5: NMR Spectra for **1e-HCl**; Figure S6: NMR Spectra for **1f**; Figure S7: NMR Spectra for **1g**; Figure S8: NMR Spectra for **2a**; Figure S9: NMR Spectra for **2b**; Figure S10: NMR Spectra for **2c**; Figure S11: NMR Spectra for **2d**; Figure S12: NMR Spectra for **2e**; Figure S13: NMR Spectra for **2f**; Figure S14: NMR Spectra for **3**; Figure S15: 2D-NMR spectra for compound **4a**; Figure S16: 2D-NMR spectra for the regioisomer of compound **4a**.

## Sample Availability

Samples of the compounds **1b**–**g**, **2a**–**f** and **3** are available from the authors.
