# Supplementary material for: Design, Synthesis and Biological Evaluation of New Pyrimidine Derivatives as Anticancer Agents
Source: Molecules. 2021 Feb 2;26(3):771. doi: 10.3390/molecules26030771 (PMC7867324; doi:10.3390/molecules26030771)
Supplement: Supplementary file 1 [file molecules-26-00771-s001.pdf]

Design, synthesis and biological evaluation of new pyrimidine derivatives as anticancer agents

Valentina Noemi Madia [1,§], Alice Nicolai [2,3,§], Antonella Messore [1,*], Alessandro De Leo [1], Davide Ialongo [1], Valeria Tudino [1], Francesco Saccoliti [4], Daniela De Vita [5], Luigi Scipione [1], Marco Artico [2], Samanta Taurone [2], Ludovica Taglieri [3], Roberto Di Santo [1], Susanna Scarpa [3], and Roberta Costi [1]

[§] These authors contributed equally

[1] Istituto Pasteur-Fondazione Cenci Bolognetti, Dipartimento di Chimica e Tecnologie del Farmaco, "Sapienza" Università di Roma, p.le Aldo Moro 5, I-00185 Rome, Italy; valentinanoemi.madia@gmail.com (V.N.M.); antonella.messore@uniroma1.it (A.M.); alessandro.deleo@uniroma1.it (A.D.L.); ialongo.1679357@studenti.uniroma1.it (D.I.); valeria.tudino@uniroma1.it (V.T.); luigi.scipione@uniroma1.it (L.S.); roberto.disanto@uniroma1.it (R.D.S.); roberta.costi@uniroma1.it (R.C.)

[2] Department of Sensory Organs, "Sapienza" University of Rome, Viale del Policlinico 155, I-00161 Rome, Italy; marco.artico@uniroma1.it (M.A.); samanta.taurone@uniroma1.it (S.T.)

[3] Department of Experimental Medicine, "Sapienza" University of Rome, Viale Regina Elena 324, I-00161 Rome, Italy; alice.nicolai@uniroma1.it (A.N.); ludovica.taglieri@yahoo.it (L.T.); susanna.scarpa@uniroma1.it (S.S.)

[4] D3 PharmaChemistry, Italian Institute of Technology, Via Morego 30, I-16163 Genova, Italy; francesco.saccoliti@iit.it

[5] Department of Environmental Biology, "Sapienza" University of Rome, p.le Aldo Moro 5, I-00185, Rome, Italy; daniela.devita@uniroma1.it

* Correspondence: antonella.messore@uniroma1.it; Tel.: +39-06-4991-3965

Contents

NMR spectra for compounds **1b-g, 2a-f** and **3**

Figure S1: NMR Spectra for **1b**;

Figure S2: NMR Spectra for **1c**;

Figure S3: NMR Spectra for **1d**;

Figure S4: NMR Spectra for **1e**;

Figure S5: NMR Spectra for **1e-HCl**;

Figure S6: NMR Spectra for **1f**;

Figure S7: NMR Spectra for **1g**;

Figure S8: NMR Spectra for **2a**;

Figure S9: NMR Spectra for **2b**;

Figure S10: NMR Spectra for **2c**;

Figure S11: NMR Spectra for **2d**;

Figure S12: NMR Spectra for **2e**;

Figure S13: NMR Spectra for **2f**;

Figure S14: NMR Spectra for **3**;

Figure S15: 2D-NMR spectra for compound **4a**

Figure S16: 2D-NMR spectra for the regioisomer of compound **4a** (namely **4a'**).

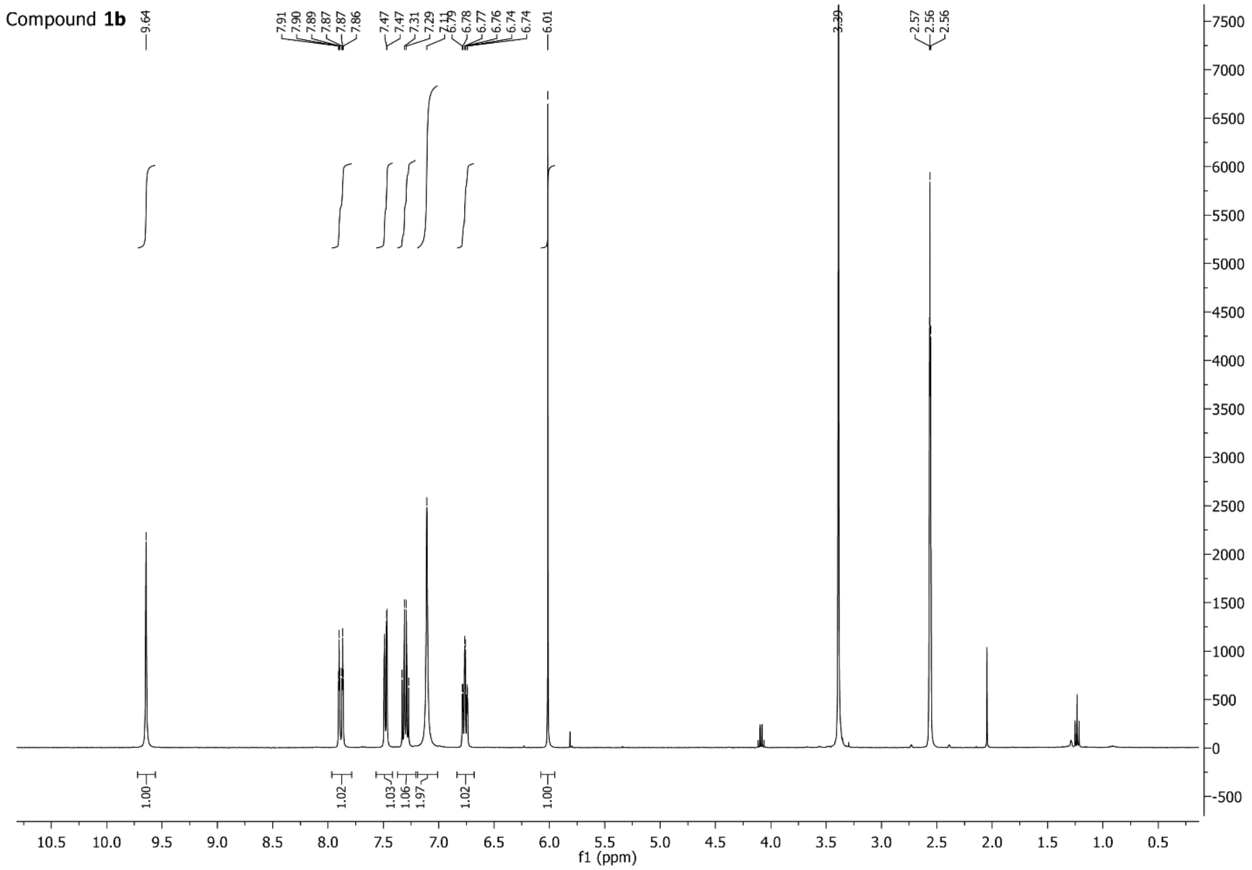

Figure S1. NMR (400 MHz, DMSO-*d*6) Spectrum for **1b**

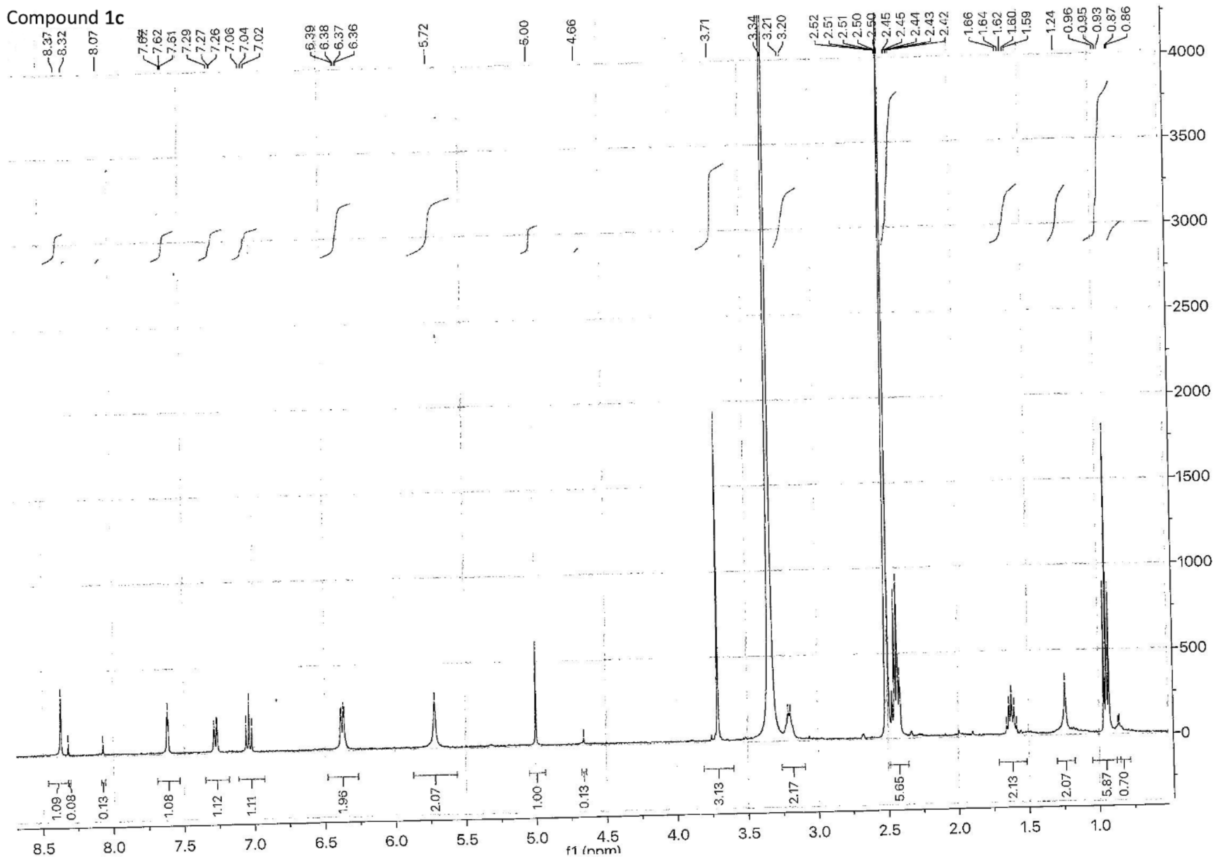

Figure S2. NMR (400 MHz, DMSO-*d6*) Spectrum for **1c**

Compound **1d**

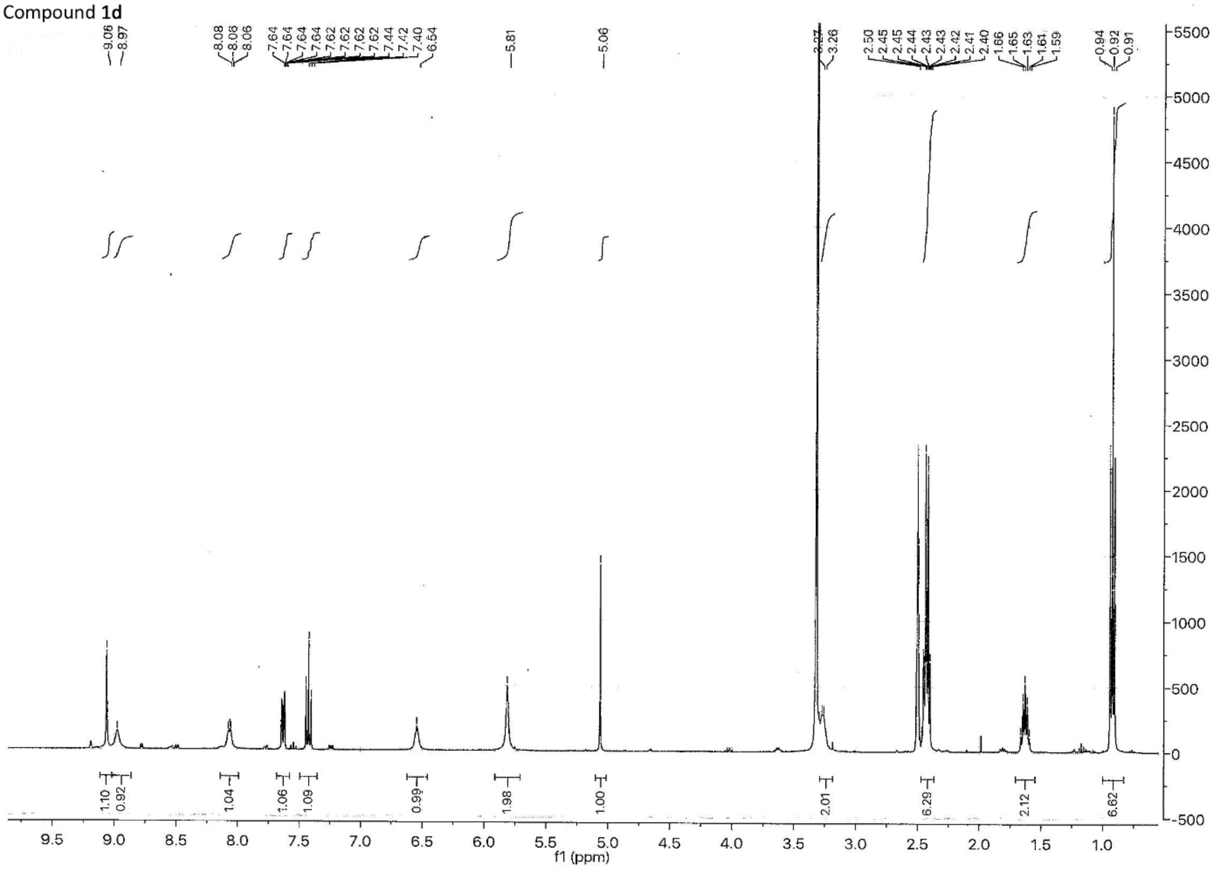

Figure S3. NMR (400 MHz, DMSO-*d*6) Spectrum for **1d**

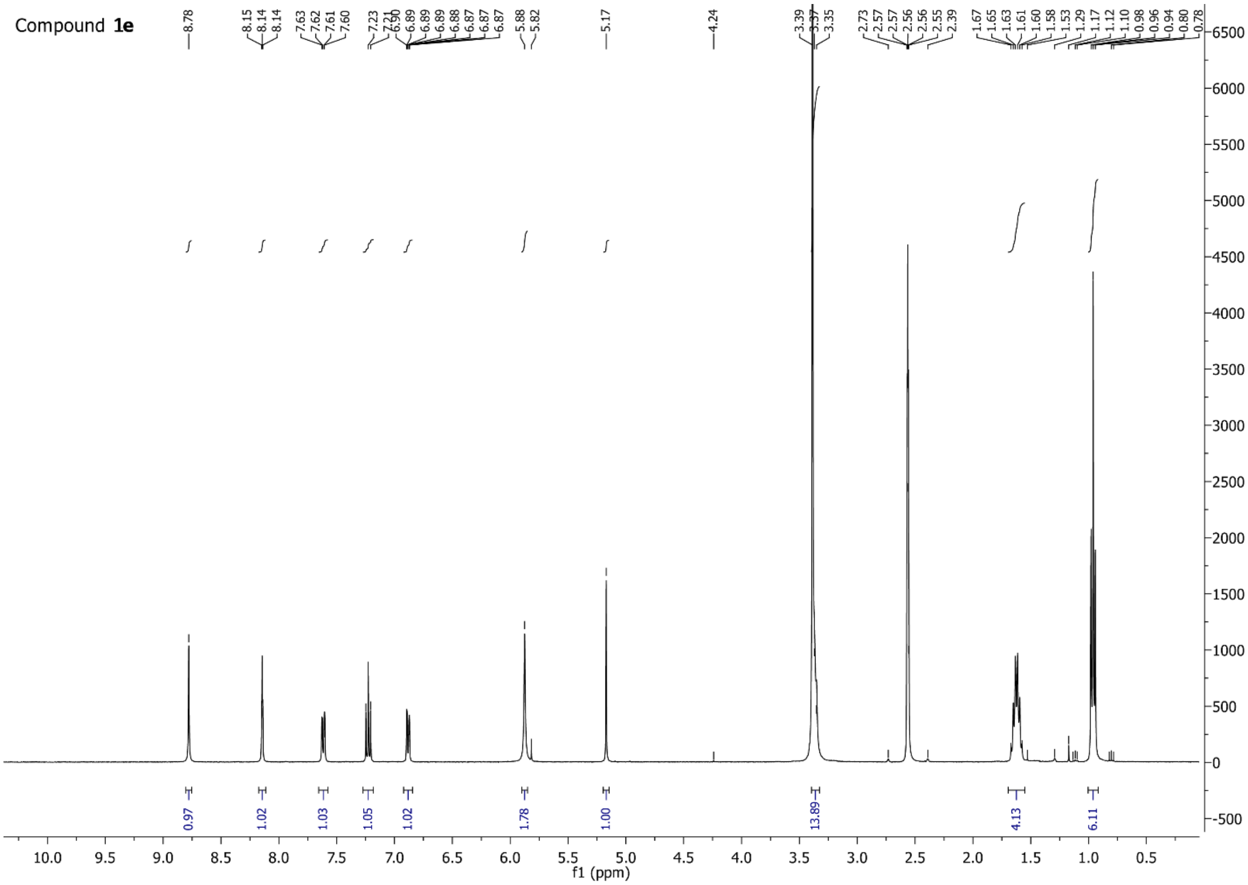

Figure S4. NMR (400 MHz, DMSO-*d*6) Spectrum for **1e**

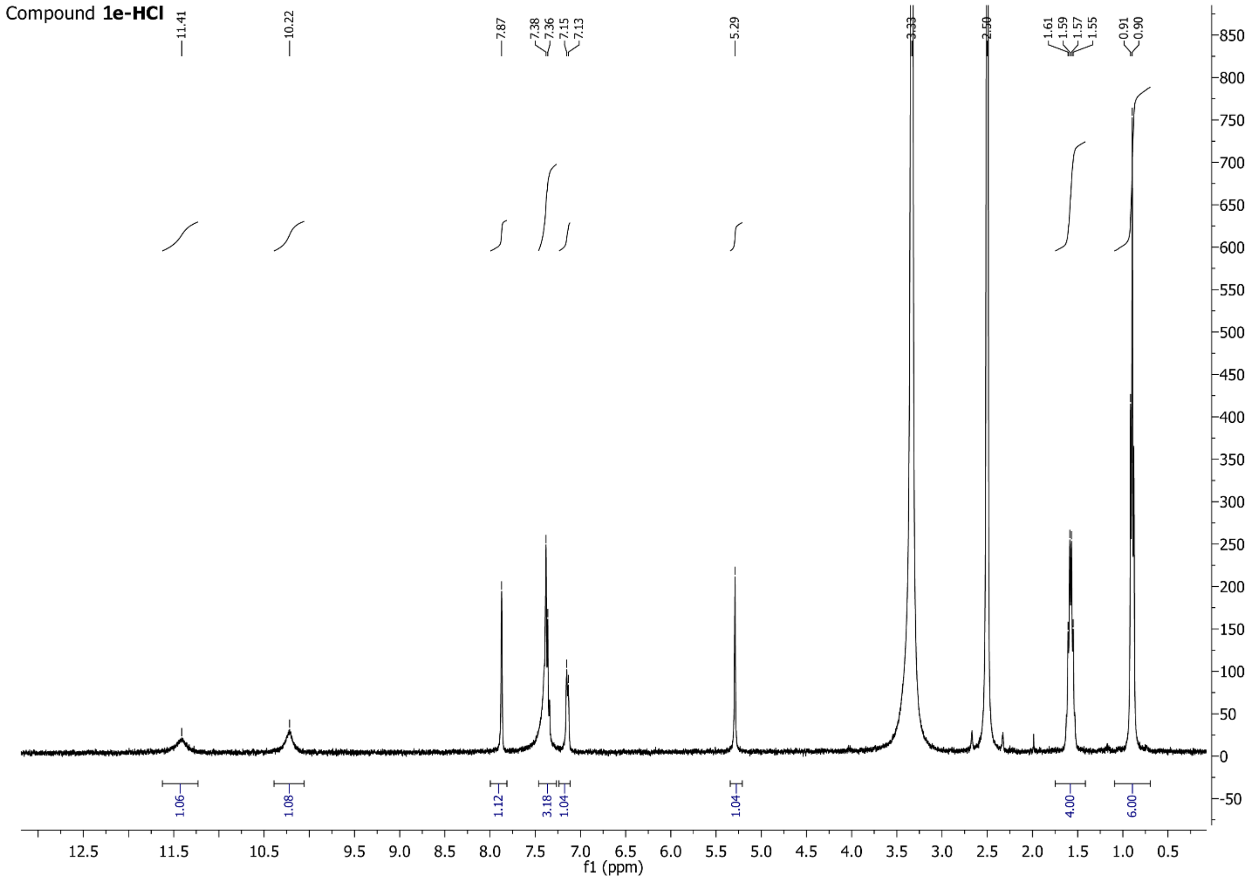

Figure S5. NMR (400 MHz, DMSO-*d*6) Spectrum for **1e-HCl**

Compound **1f**

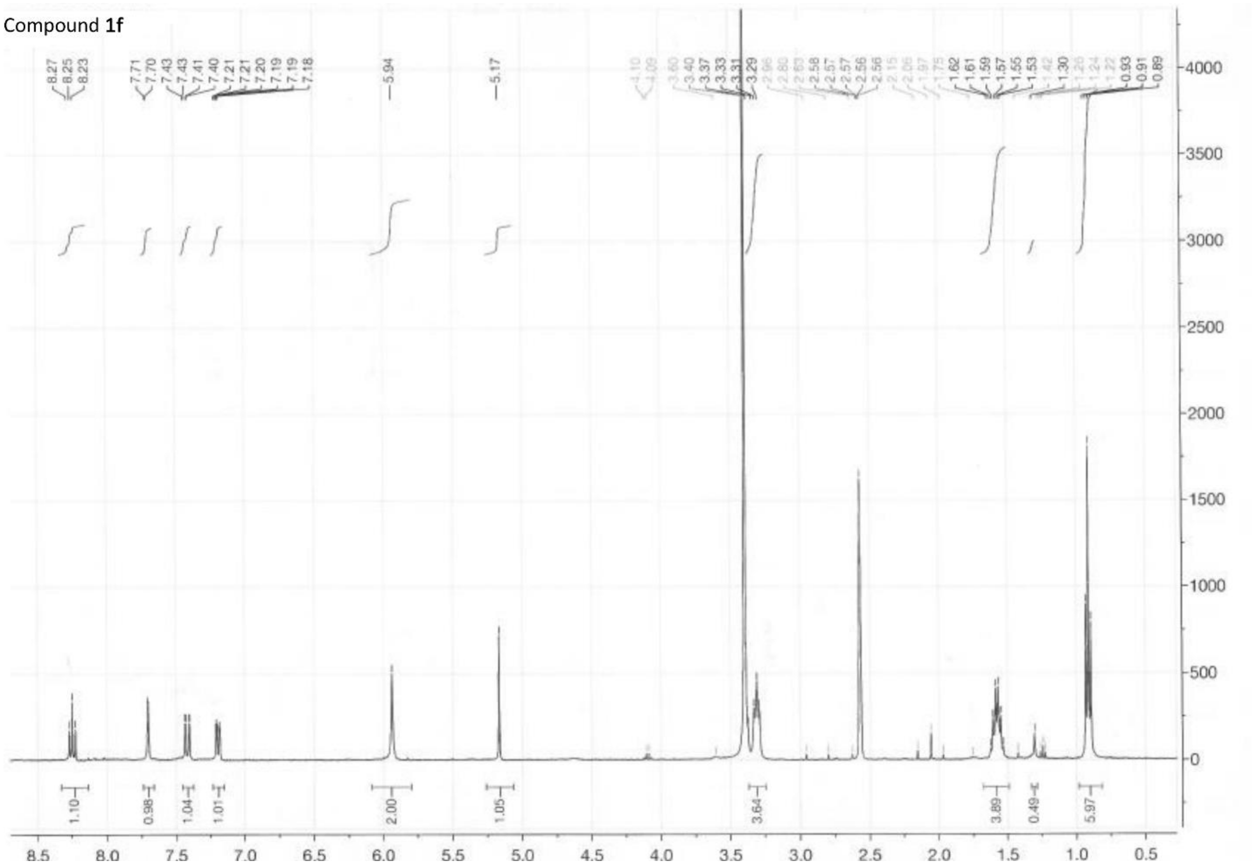

Figure S6. NMR (400 MHz, DMSO-*d*6) Spectrum for **1f**

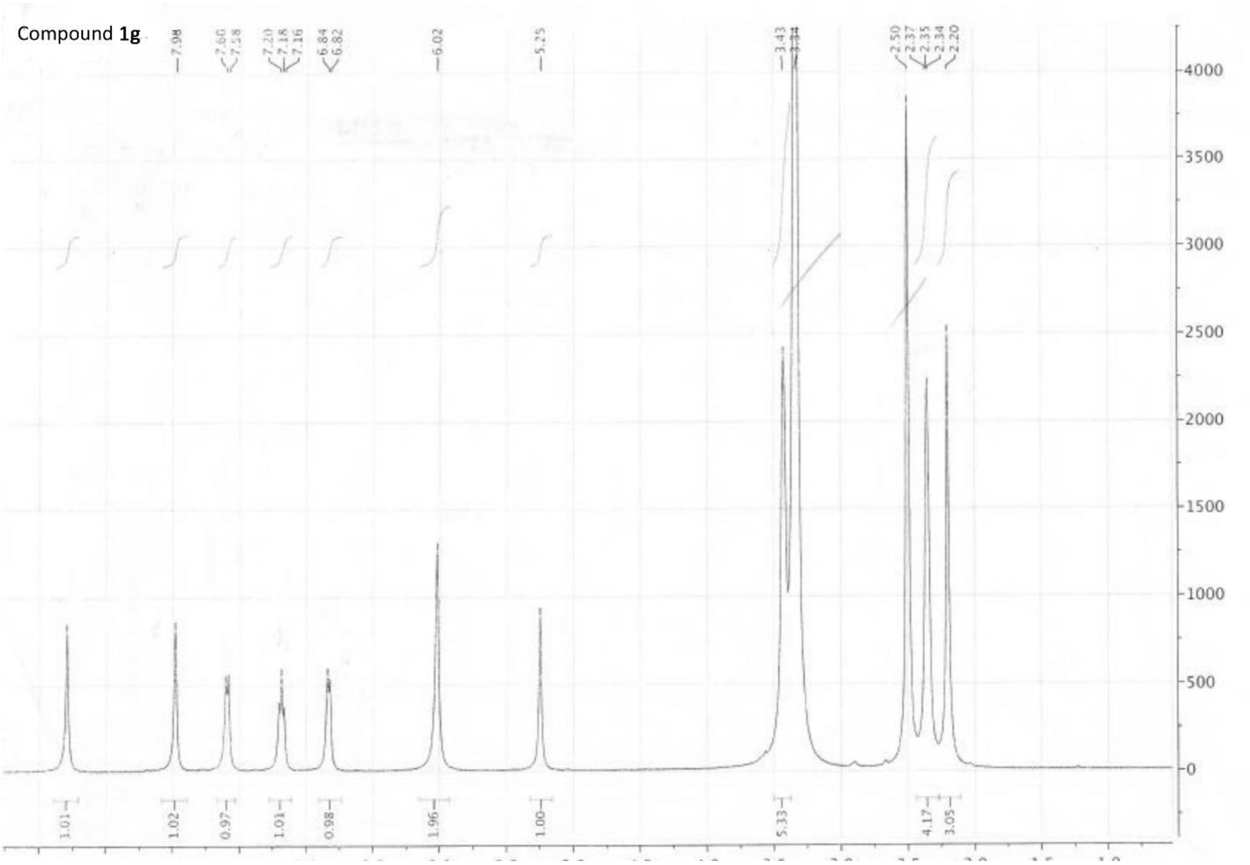

Figure S7. NMR (400 MHz, DMSO-*d*6) Spectrum for **1g**

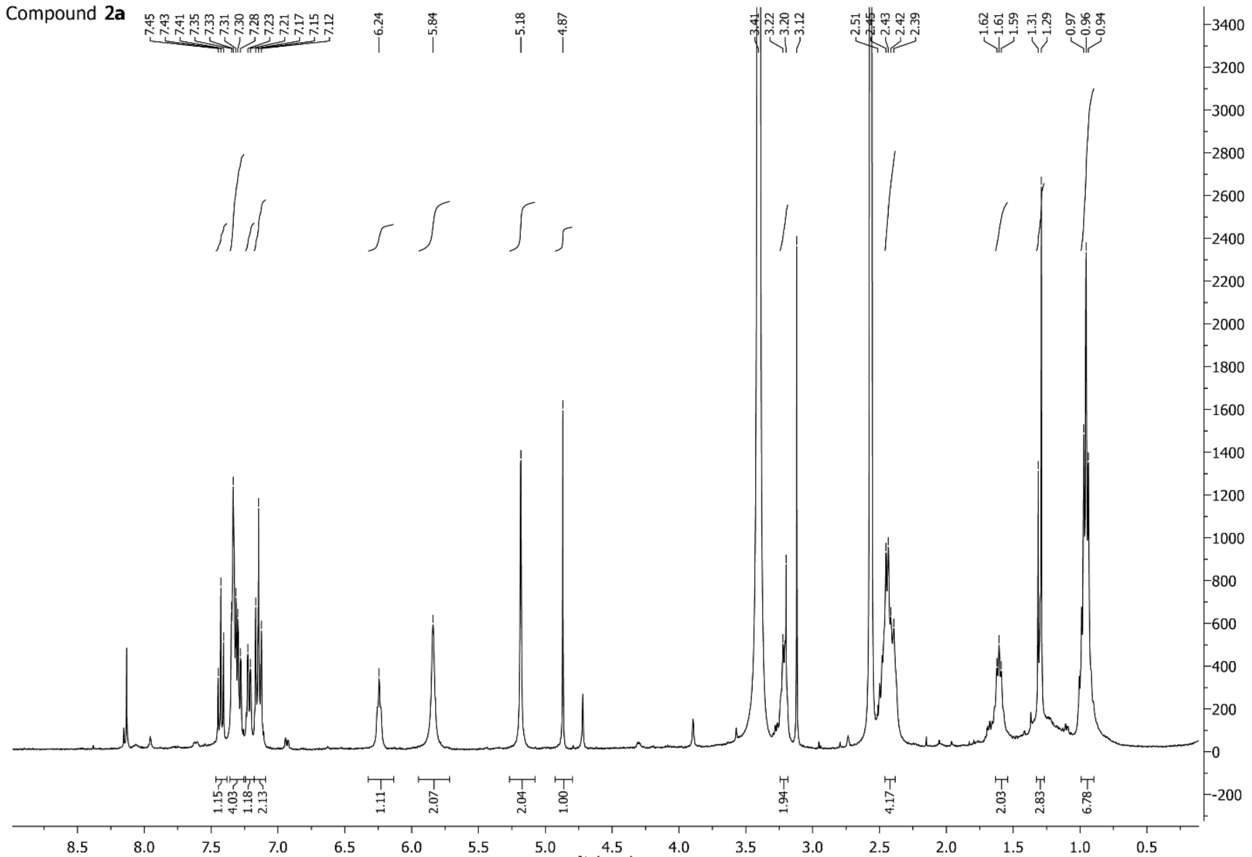

Figure S8. NMR (400 MHz, DMSO-*d*6) Spectrum for **2a**

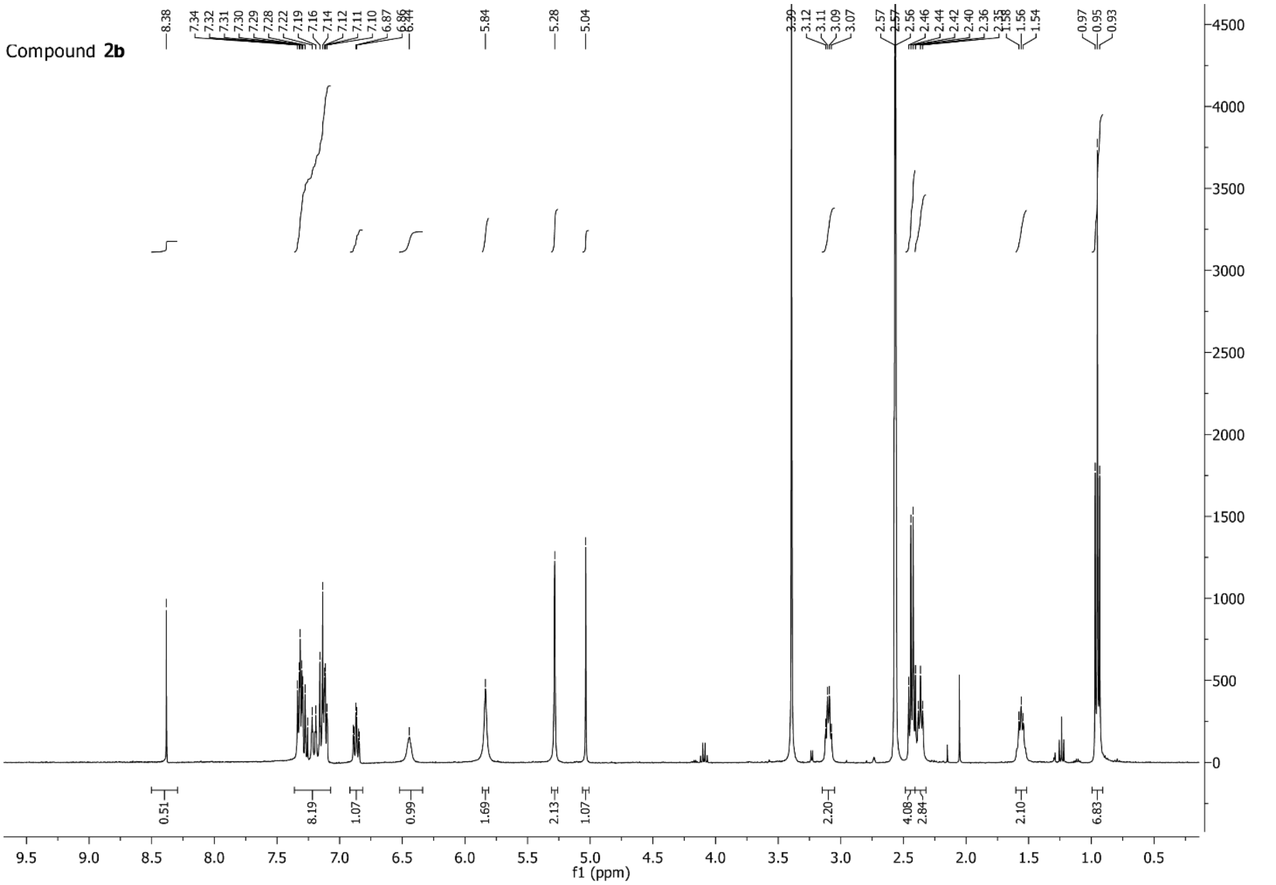

Figure S9. NMR (400 MHz, DMSO-*d*6) Spectrum for **2b**

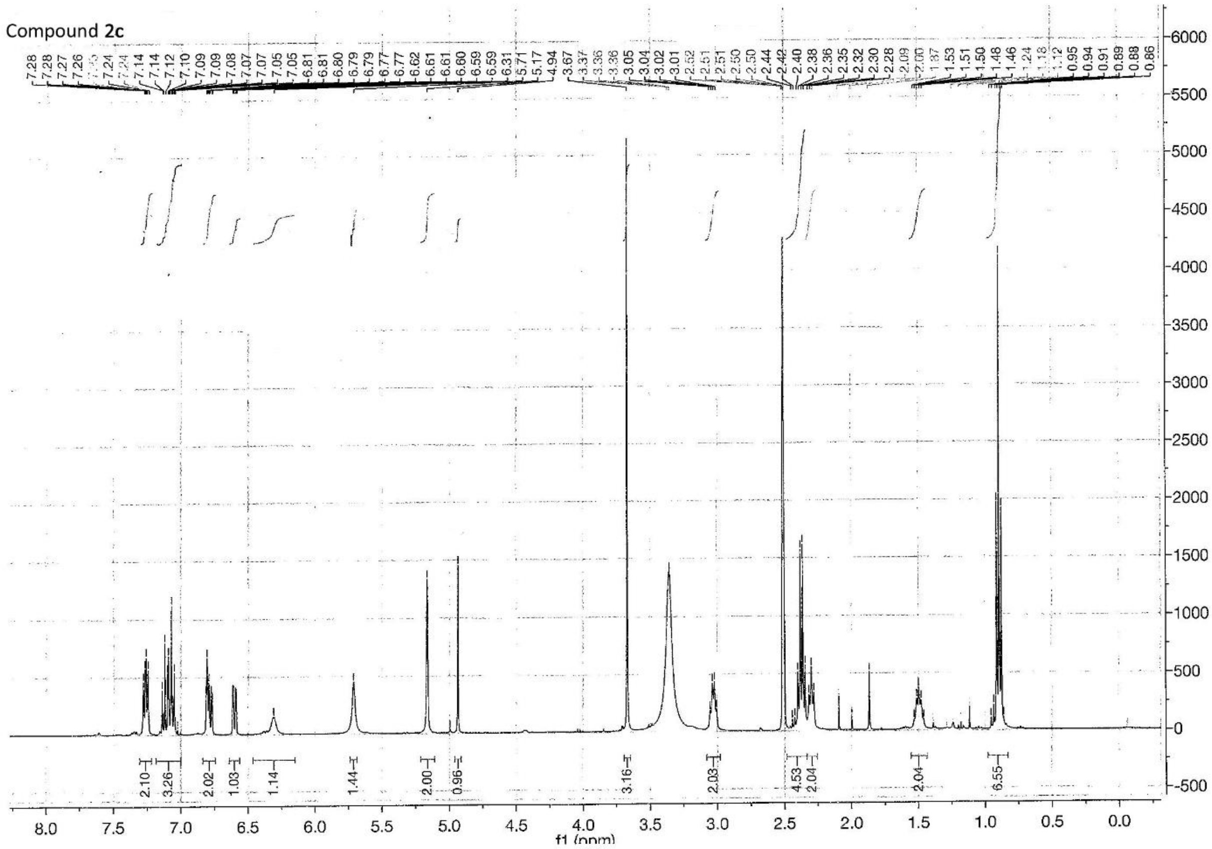

Figure S10. NMR (400 MHz, DMSO-*d*6) Spectrum for **2c**

Compound **2d**

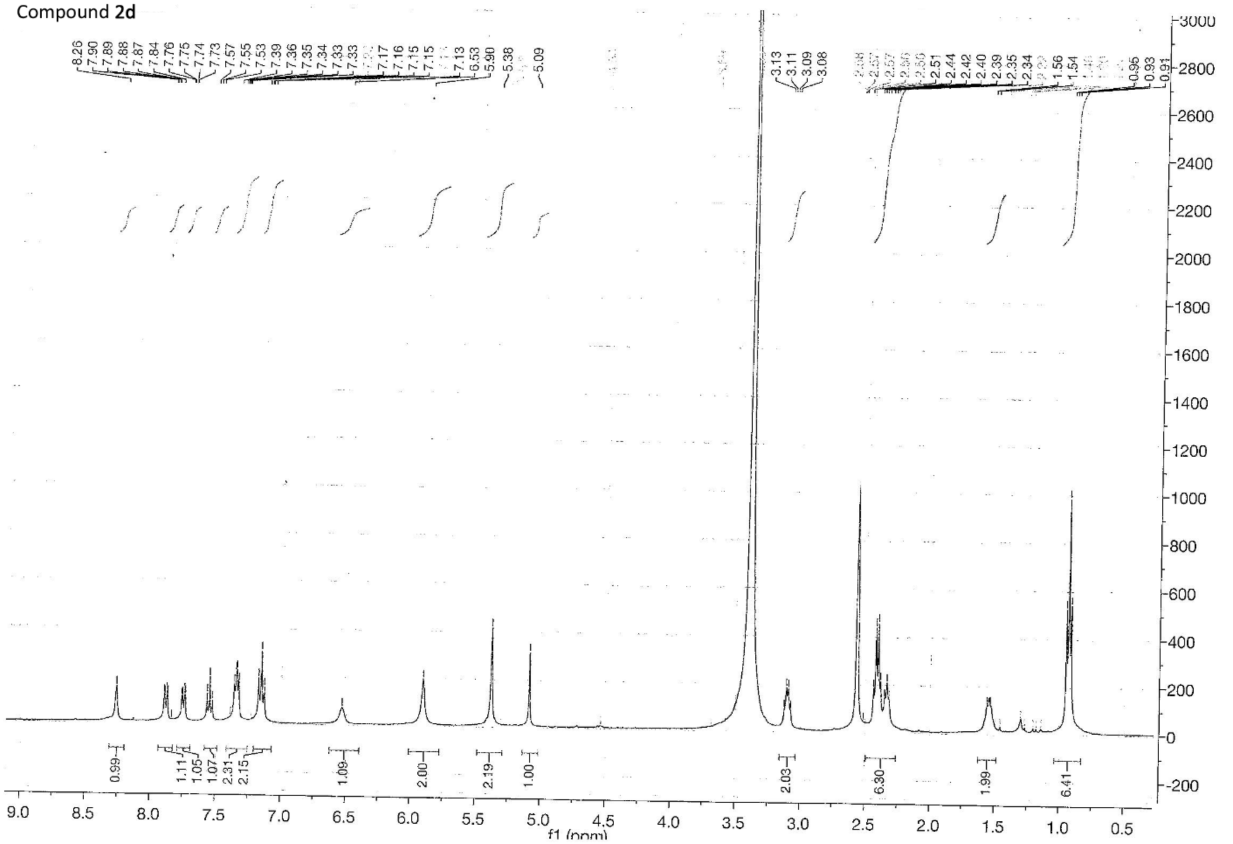

Figure S11. NMR (400 MHz, DMSO-*d*6) Spectrum for **2d**

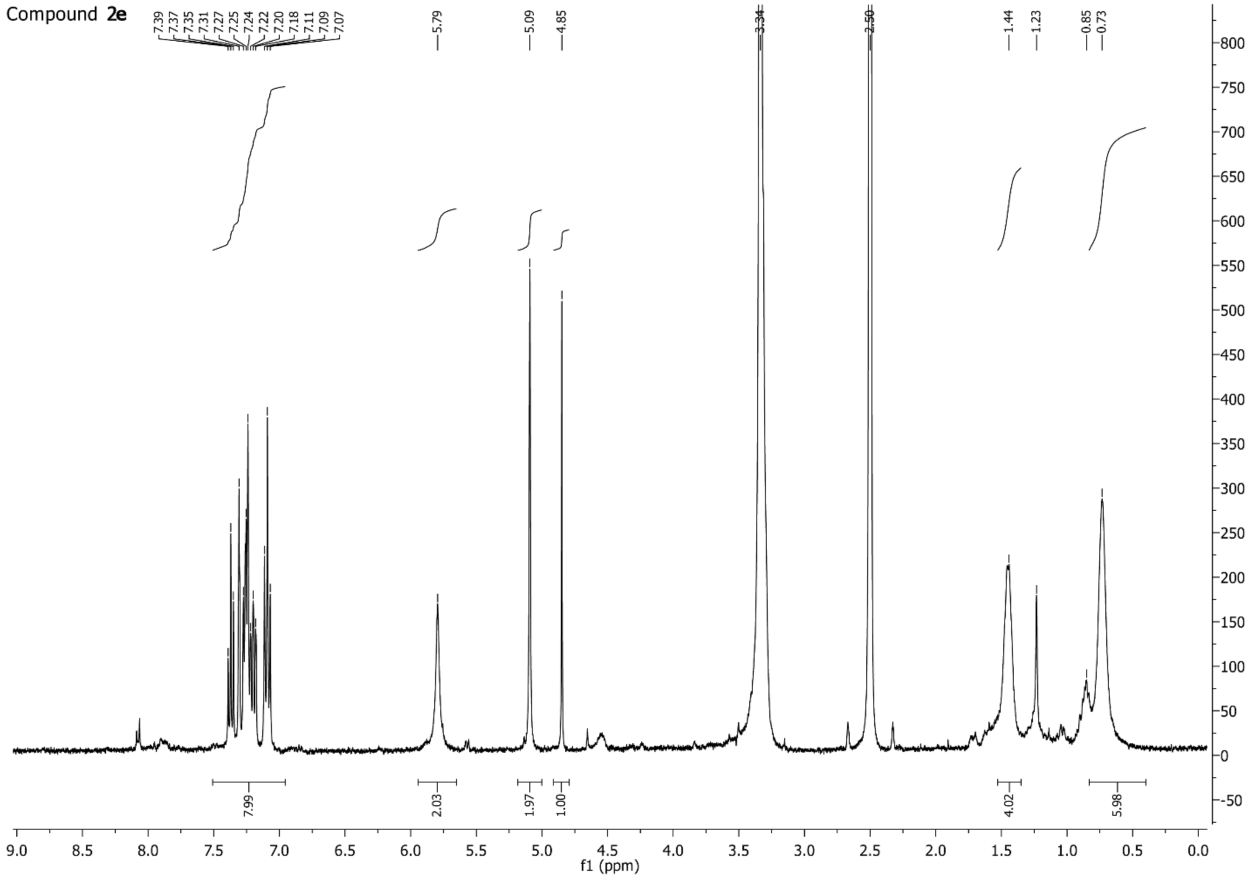

Figure S12. NMR (400 MHz, DMSO-*d6*) Spectrum for **2e**

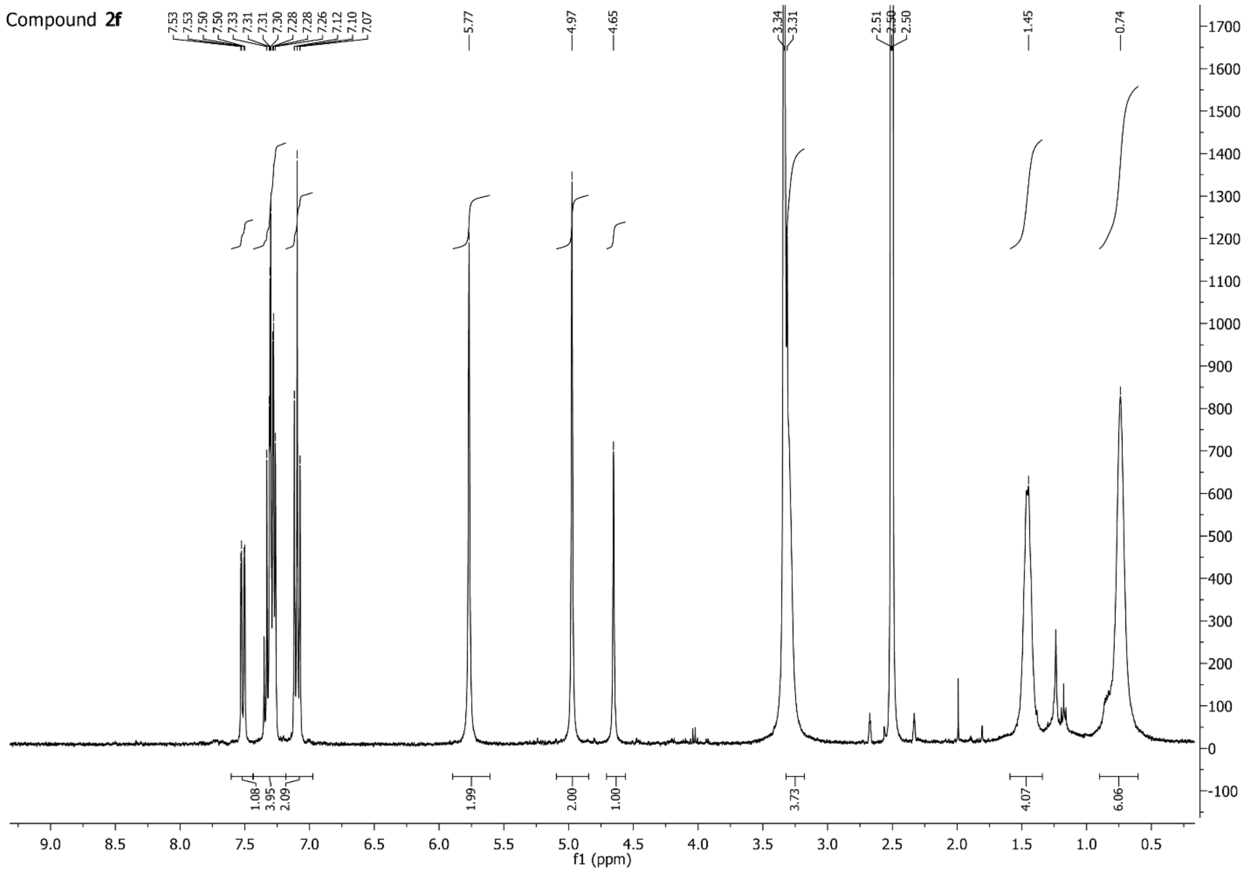

Figure S13. NMR (400 MHz, DMSO-*d*6) Spectrum for **2f**

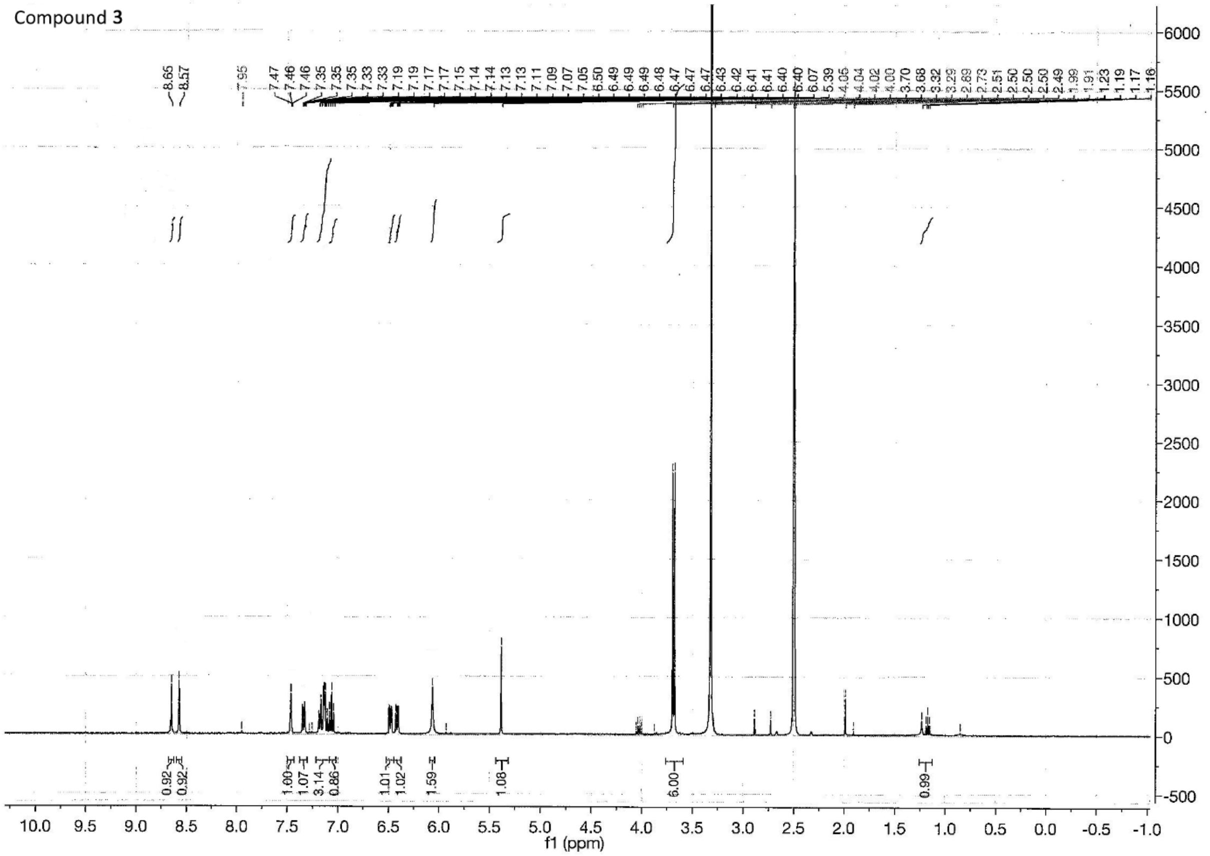

Figure S14. NMR (400 MHz, DMSO-*d*6) Spectrum for **3**

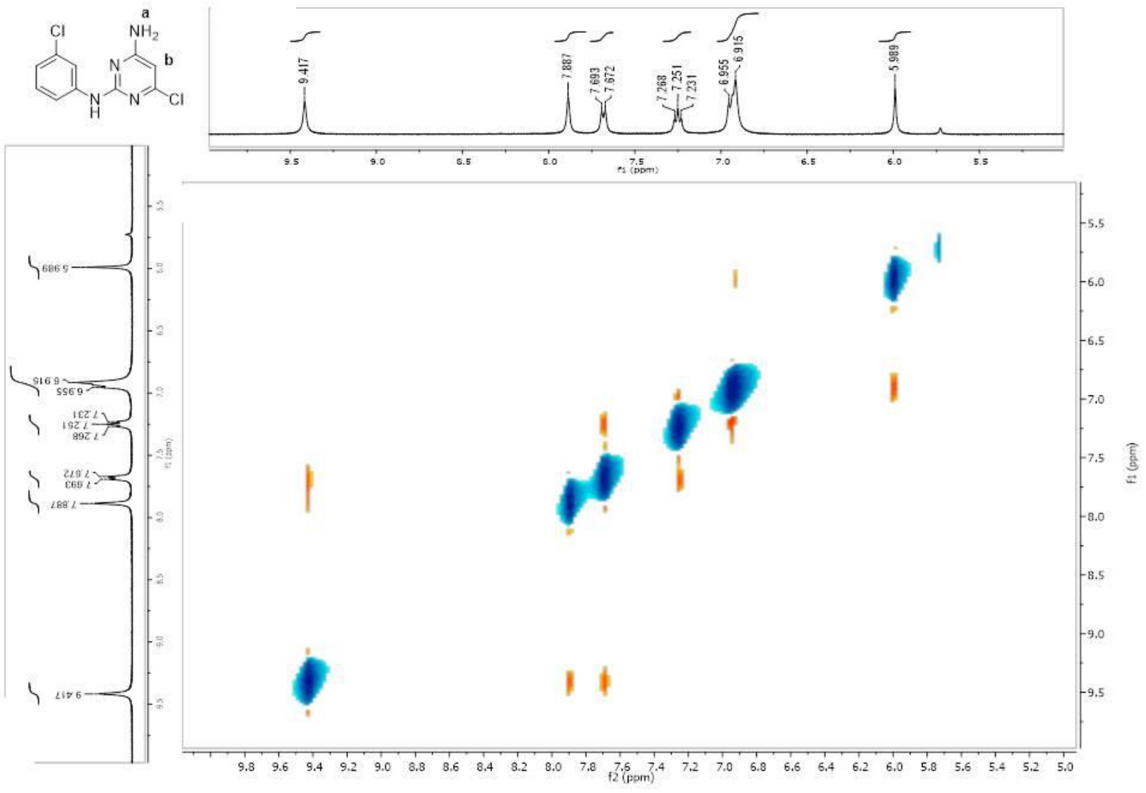

Figure S15. 2D-NMR (400 MHz, DMSO-*d*6) Spectrum for **4a**. The singlet at 5.98 ppm correlates with the broad singlet at 6.90 ppm: these signals correspond respectively to Hb and NH_2 of pyrimidine moiety. The cross peak indicates the closeness of Hb to NH_2 and confirms the structure reported for compound **4a**

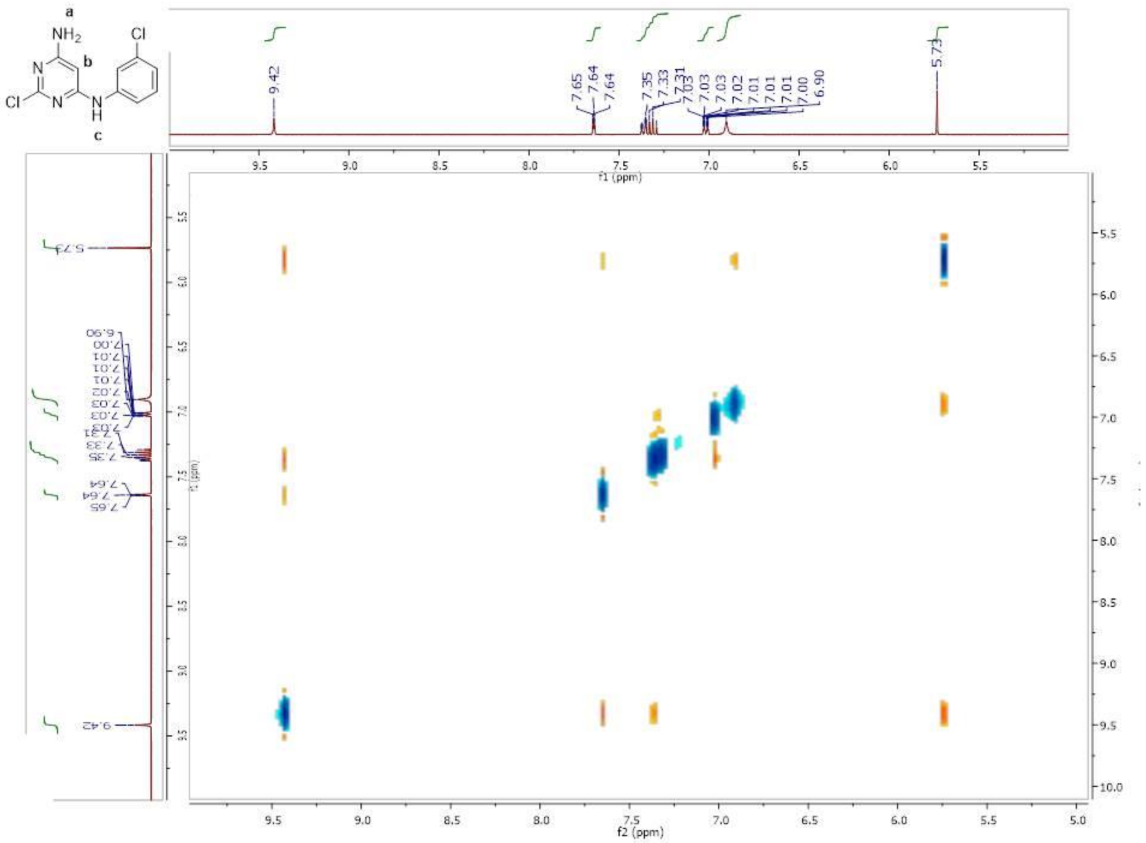

Figure S16. 2D-NMR (400 MHz, DMSO-*d*6) spectra for the regioisomer of compound **4a** (namely **4a'**). The singlet at 5.78 ppm correlates with the broad singlet at 6.90 ppm and with the singlet at 9.42 ppm: these signals correspond respectively to Hb and NH_2 of pyrimidine moiety and NH (Hc) of the aniline portion. In this spectrum there are NOE correlations between the hydrogens of pyrimidine moiety and the Hc of the aniline portion and this confirms the structure reported for compound **4a'.**
